# In “Tone” with dogs: exploring canine musicality

**DOI:** 10.1007/s10071-024-01875-5

**Published:** 2024-05-16

**Authors:** Claudia Pinelli, Anna Scandurra, Cristina Giacoma, Alfredo Di Lucrezia, Biagio D’Aniello

**Affiliations:** 1 Department of Environmental, Biological and Pharmaceutical Sciences & Technologies, University of Campania “Luigi Vanvitelli”, Caserta, 81100 Italy; 2 Department of Biology, University of Naples Federico II, Naples, 80126 Italy; 3 Department of Life Sciences and System Biology, University of Torino, Torino, 10123 Italy

**Keywords:** Dogs, Musicality, Domestication, Absolute pitch, Relative pitch, Cognition

## Abstract

This study investigates the musical perception skills of dogs through playback experiments. Dogs were trained to distinguish between two different target locations based on a sequence of four ascending or descending notes. A total of 16 dogs of different breeds, age, and sex, but all of them with at least basic training, were recruited for the study. Dogs received training from their respective owners in a suitable environment within their familiar home settings. The training sequence consisted of notes [Do-Mi-Sol#-Do (C7-E7-G7#-C8; Hz frequency: 2093, 2639, 3322, 4186)] digitally generated as pure sinusoidal tones. The training protocol comprised 3 sequential training levels, with each level consisting of 4 sessions with a minimum of 10 trials per session. In the test phase, the sequence was transposed to evaluate whether dogs used relative pitch when identifying the sequences. A correct response by the dog was recorded as 1, while an incorrect response, occurring when the dog chose the opposite zone of the bowl, was marked as 0. Statistical analyses were performed using a binomial test. Among 16 dogs, only two consistently performed above the chance level, demonstrating the ability to recognize relative pitch, even with transposed sequences. This study suggests that dogs may have the ability to attend to relative pitch, a critical aspect of human musicality.

**Supplementary Information:**

The online version contains supplementary material available at 10.1007/s10071-024-01875-5.

## Introduction

Musicality is an innate trait related to both biological factors and cognitive processes (Honing et al. 2015). Musicality in humans is a complex and multifaceted concept that encompasses both inherent and learned abilities to understand, appreciate, and create music. It involves a wide range of skills, including the perception of rhythm, pitch, melody, harmony, and timbre of musical components. Musicality extends beyond technical proficiency encompassing sensitivity to the emotional and aesthetic aspects of music, which are universally present in various human cultures (Brown and Jordania 2011).

Many species exhibit behaviors that can be compared to human music-making, which serves as a means of communication. While birdsongs are the most obvious example (Rothenberg et al. 2014), whale and seal songs have also been considered musical expressions (Payne and McVay 1971). In addition, it is thought that certain non-vocal sounds, such as the rhythmic drumming on the chest or tree buttresses by primates (Babiszewska et al. 2015) and woodpeckers on tree bark (Dodenhoff et al. 2001) can convey musical elements. Certain species exhibit a so highly developed cognitive skills for musicality that enables them to engage in duetting (Hall 2009; Torti et al. 2013) or imitate the tones (Williams 1990; Nottebohm 1992; Abramson et al. 2018). Additionally, some species have shown the ability to discriminate between regular (isochronous) and irregular (non-isochronous) sequences of the same notes by discerning rhythmic patterns (e.g., rats: Celma-Miralles and Toro 2020; starlings: Hulse et al. 1984; Humpal and Cynx 1984; jackdaws: Reinert 1965) and some species produce vocalizations characterized by consistent and recognizable patterns of timing and frequency **(**vocal categorical rhythms) similar to the rhythmic patterns observed in human speech and communication (De Gregorio et al. 2021).

To explore the cognitive aspects of musicality in non-human animals, researchers employ various methodological approaches. One such approach involves observing the phenomenon of motor entrainment to rhythmic sounds. This includes the synchronization of one’s movements with a rhythmic stimulus, such as nodding one’s head or tapping one’s foot to music. The observation of whether animals can synchronize their movements to a musical beat provides insight into their ability to perceive and process rhythms and melodies. Research has demonstrated that certain species of parrots and sea lions can synchronize their movements as the music tempo is experimentally manipulated (Patel et al. 2009; Schachner et al. 2009; Cook et al. 2013; Rouse et al. 2016).

Musicality also encompasses the ability to recognize octave equivalence, a concept indicating that notes separated by an octave appear more similar than notes closer in frequency. This ability has been observed in both children and adults, as evidenced by young children spontaneously transposing melodies sung by adults outside their vocal range by an octave (Hoeschele 2017). Interestingly, a female bottle-nosed dolphin also exhibited this ability by octave-transposing sounds outside her preferred vocal range (Richards et al. 1984). While octave equivalence has been observed in rhesus macaques, studies on birds have not provided conclusive results (Cynx 1993; Hoeschele et al. 2013; Wagner et al. 2019; Wagner and Hoeschele 2022).

There has been a paucity of research on the musical aptitudes of species that have adapted to thrive in the anthropogenic niche, creating a distinctive ecological and evolutionary backdrop that might affect their auditory perception and processing. Dogs, which have a particularly close association with humans, are one such species that have been relatively underexplored concerning musicality.

Researchers have investigated whether dogs are responsive to music and if it elicits any emotional changes in them. The findings indicate that certain types of music, such as classical compositions or soft melodies, can have a calming effect on dogs, while high-pitched, or overly loud music can agitate dogs and increase their anxiety or restlessness (Lindig et al. 2020). However, the emotional effects of music on dogs do not necessarily imply that they have a cognitive skills for musicality. Moreover, studies suggest that dogs may not be capable of motor entrainment (Schachner et al. 2009).

In the early 1900s, some authors conducted experiments to examine auditory discrimination in dogs. These experiments included playing various notes on instruments or producing sounds from different whistles. They found that dogs could discriminate between them (Kalischer 1907; Selionyi 1907), but it was not clear whether this ability was related to the timbre of the instruments or different notes being played at the same pitch. Later, Shepherd (1919) conducted experiments in which two dogs were trained to distinguish between two notes with the same pitch class (i.e., octave transpositions) and the same timbre. One dog was successful in mastering the task after 300 trials when there was a three-octave difference in pitch between the learned and the transposed note. However, when tested on discrimination of transposed notes occurring within a single octave, the dog did not perform as well. The other dog showed no clear indication of discrimination. The latter research revealed that at least one dog had the ability to “distinguish octave equivalence”, i.e., perceive and recognize different notes with the same pitch class. However, musicality requires an understanding of the relationships and interplay between sounds, which cannot be inferred from the experiments described, as only a single note was used.

Hence, at present, there is a lack of definitive evidence indicating whether dogs have aspects of musicality. Nevertheless, rhythmic information in barks or pitch in howls could be relevant for dogs. Moreover, living near humans and being exposed to a musical environment for several generations might have prompted the development of some of these abilities.

In the current paper, we aimed to investigate dogs’ capacity to process relative pitch. To achieve this goal, we conducted a series of playback experiments using a consistent sequence of notes requiring dogs to reach different target locations. The subjects that successfully mastered the task during training were included in the testing procedure, where they were presented with transposed sequences of the learned ones. This method allowed us to assess whether dogs could memorize sound sequences and whether they use relative pitch to do so.

## Materials and methods

The study aimed to train dogs to reach two different target locations based on a specific sequence of notes. The sequence of notes remained consistent throughout the training but was played in either an ascending (from the lowest to the highest sound) or descending (from the highest to the lowest sound) scale. The dogs underwent training sessions to learn the associations between the ascending or descending sequence and the corresponding target locations. If the dogs successfully mastered the task during training, they were then admitted to the testing procedure during which the sequences were transposed, i.e., the original sequence was shifted by 6 (half octave), 12 (one octave), 18 (one octave and half), or 24 (two octaves) notes, either up or down, relative to the training sequence.

### Participants

A total of 16 dogs of different breeds, age and sex but all of them with at least basic training were recruited for the study (see Online Resource 1). Twelve of these dogs received training from their respective owners in a suitable environment within their familiar home settings. Among the participants, three dogs belonged to the authors of the current paper. Additionally, one of the authors, although not the owner, trained another dog in the laboratory. It is worth noting that thirteen of the owners were certificated dog trainers, including one of the authors.

### Musical sequences

**Table 1:** Musical notes and the corresponding frequencies of the sequences used in the study

-24	Hz	-18	Hz	-12	Hz	-6	Hz	0	Hz	+6	Hz	+12	Hz	+18	Hz	+24	Hz
Do (C5)	523	Fa# (F5#)	740	Do (C6)	1047	Fa# (F6#)	1480	Do (C7)	2093	Fa# (F7#)	2962	Do (C8)	4186	Fa# (F8#)	5921	Do (C9)	8372
Mi (E5)	660	La# (A5#)	932	Mi (E6)	1319	La# (A6#)	1866	Mi (E7)	2639	La# (A7#)	3730	Mi (E8)	5275	La# (A8#)	7458	Mi (E9)	10,548
Sol# (G5#)	832	Re (D6)	1176	Sol# (G6#)	1662	Re (D7)	2350	Sol# (G7#)	3322	Re (D8)	4698	Sol# (G8#)	6646	Re (D9)	9396	Sol# (G9#)	13,298
Do (C6)	1047	Fa# (F6)	1480	Do (C7)	2093	Fa# (F7)	2962	Do (C8)	4186	Fa# (F8#)	5921	Do (C9)	8372	Fa# (F9#)	11,839	Do (C10)	16,745

Level 0 (dark gray column) indicates the note sequence used for training. The numbers in the top row (+/-6; +/-12; +/-18; +/-24) indicate the transposition of the first note in the respective test sequences (medium grey and light grey columns) by 6 (half octave), 12 (one octave), 18 (one octave and half), or 24 (two octaves) notes, either up (+) or down (-) relative to the training sequence. Hz = frequency of notes in hertz. Letters in brackets = indication of notes in Scientific Pitch Notation (SPN), where “Do” corresponds to the note “C”. Note that, 12 and 24 transpositions encompass the same notes, while 6 and 18 transpositions involve distinct notes

During the training phase of the study, the dogs were exposed to a specific sequence of notes. These notes were digitally generated using the free open-source software Audacity 2.4.2 and produced as pure sinusoidal tones. A consistent temporal pattern was employed for both the duration of the tones (245 msec) and the intervals between the tones (100 msec). The overall duration of the stimulus was 1.480 s. The sequence was presented to the dogs in ascending or descending order. The ascending sequence was played in the following order: Do-Mi-Sol#-Do (C7-E7-G7#-C8; Hz frequency: 2093, 2639, 3322, 4186); the descending sequence was: Do-Sol#-Mi-Do (C7-G7#-E7-C8; Hz frequency: 4186, 3322, 2639, 2093) (Table 1).

In the testing phase, the same temporal pattern was maintained, but transpositions were introduced. The original sequence was transposed by a tritone (half octave), 12 (one octave), 18 (one octave and half) and 24 (two octaves) notes higher (+) and lower (-) from the initial Do (C7). In the transpositions of 6 and 18 notes, despite altering the initial note (i.e., Fa# (F#) instead of Do (C)) while maintaining the same melodic pattern, the arrangement was designed to prevent the dogs from relying solely on single tone recognition. This approach aimed to ensure that their learning focused on the melody of the whole sequence of notes and the pattern they formed.

To avoid potential bias arising from reinforcement-induced learning during testing phases, we examined the first responses of the dogs to all transposed sequences. Analysing these responses, where the dogs encountered the new sequence for the first time and therefore couldn’t rely on prior reinforcement, aimed to confirm that their reactions were not simply repetitions of quickly learned behaviors induced by the reward.

To avoid consecutive repetitions, the ascending or descending sequence was presented to the dogs in a randomized manner, ensuring that the same sequence was not played consecutively more than twice. Each presentation of a sound stimulus to the dogs, whether ascending or descending, was separated by a variable interval. This interval corresponded to the time required for the owner to return the dog to the starting position and regain the dog’s attention.

### Procedure

A long preliminary phase was necessary to develop an appropriate experimental protocol involving a considerable number of dogs, evaluation of training techniques, variations of tone sequences, target locations, and the overall structure of the experimental setting.

**Fig. 1:**
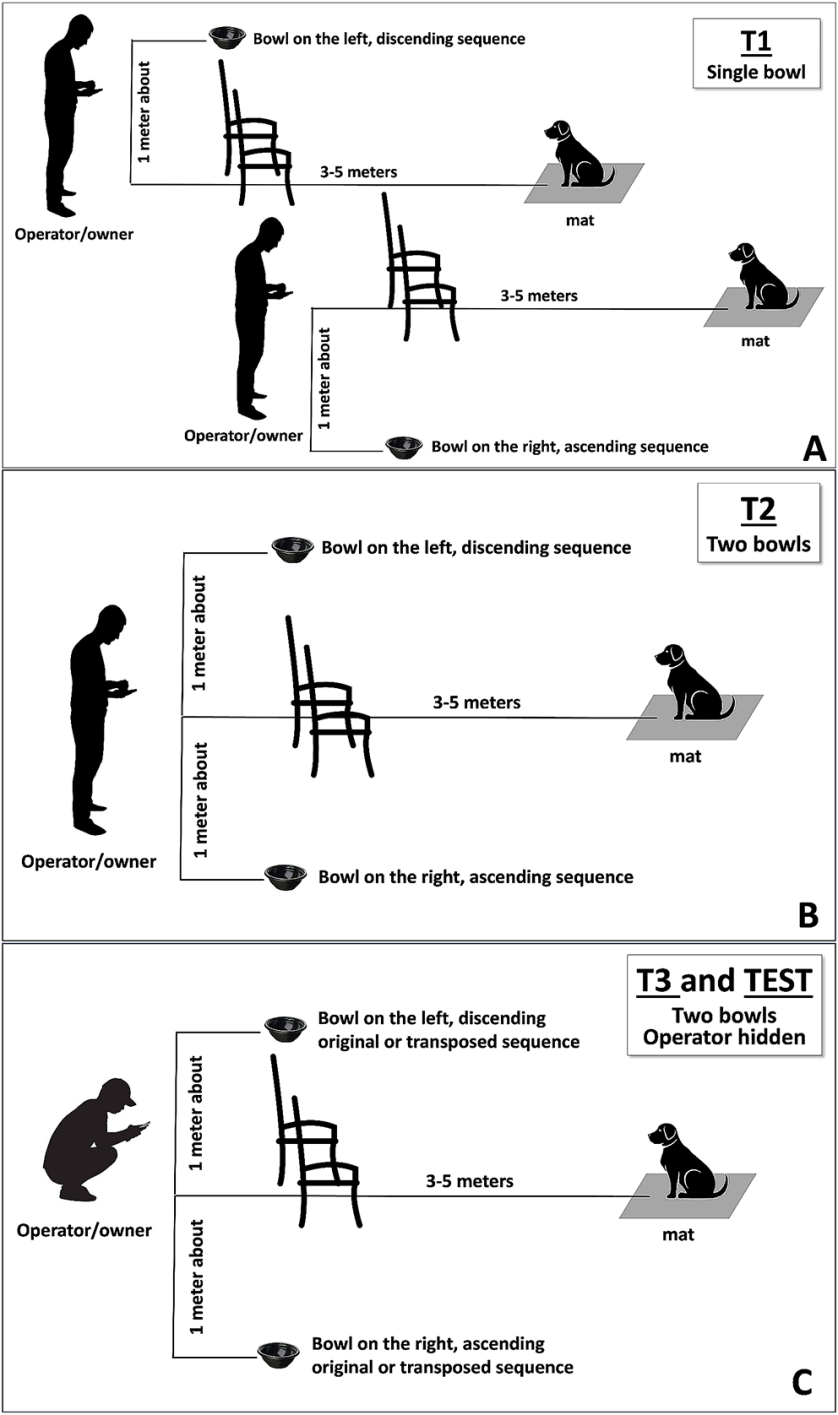
The experimental setting in training levels (T) and the testing pahse (TEST). (**A**) T1. Only one bowl was used. The dog was on a mat 3–4 m from the barriers in the rest position. The bowl on the left side of the operator was reserved for the descending sequence, while that on the right side was for the ascending sequence. (**B**) T2. The configuration was the same as T1, but a bowl was placed on either side of the owner. (**C**) T3 and testing phase (TEST). The configuration was the same as T2, but the owner was hidden behind the barrier

The selected training protocol comprised 3 sequential training levels (Fig. 1), with each level consisting of 4 sessions with a minimum of 10 trials per session. Participants were asked to conduct not more than 2 or 3 sessions per week. The authors closely monitored the training levels by reviewing each video before proceeding to the next session or level. They occasionally provided suggestions to enhance the training.

In Training level 1 (T1; Fig. 1A), owners utilized either chairs covered by blankets or an armchair as barriers to conceal themselves up to the waist. This setup was designed to completely conceal the owner during Training level 3 (see below). A mobile phone was placed on a chair positioned behind the dog to record the trials. In some sessions, a familiar person recorded the videos. In the laboratory setting, a wooden panel served as barrier, and a closed-circuit television system equipped with four cameras was employed for recording purposes. The owners instructed the dog to sit on a mat and maintain a resting position while reaching the area beyond the barriers (the dogs selected for the experiment were already trained to respect the “rest” command). The dog sat approximately 3–4 m in front of the owner. For the ascending sequence, a bowl was placed on the right side of the owner, while the other bowl was not present. Conversely, for the descending sequence, only the bowl on the left side was available. The owners were instructed to avoid direct eye contact with the dogs but instead focus on their mobile phones. Once the owners played the sequences on their mobile phones, they employed verbal and physical encouragement to prompt the dog to move towards the requested side. Once the dog reached the correct side, a piece of sausage was placed inside the bowl by the owner as a reward. However, if the dog remained in its resting position for more than three seconds after the sequence had been played, the sequence was replayed, and the dog was encouraged once again to reach the target location. Conversely, if the dog left the resting position before the owner played the sequence or exited the experimental area, the trial was considered null. During the initial trials, the owners were allowed to assist the dog using body movements and pointing gestures if necessary. However, as the trials progressed and based on a sequence of correct responses, the researchers suggested the owners to eliminate the use of body language. A correct response by the dog was recorded as 1, while an incorrect response, which occurred when the dog chose the opposite zone of the bowl, was marked as 0.

Training level 2 (T2; Fig. 1B) followed a similar procedure to T1, with the exception that a bowl was placed on either side of the owner. Most of the dogs underwent the four training sessions preconfigured, which constituted the “*short-training*” duration. However, for three dogs, a longer training period was implemented, consisting of 24, 25, and 29 sessions, respectively. This “*long-training*” aimed to ensure that any failure to master the task was not due to a limited number of trials. In T2, wrong responses were defined as choosing the incorrect target location.

In Training level 3 (T3; Fig. 1C), a bowl was placed on either side of the owner like in T2, but the owner was invisible to the dogs as he hid behind the barrier. However, in some cases the owner still had the opportunity to observe and control the dog’s behavior during the trial by looking through a small hole or space located at the center of the barrier. This training level aimed to control for any “Clever Hans” effect.

If the cumulative number of successful trials in all the sessions included in a training level was statistically above the chance level based on a binomial test, the dog was deemed eligible for the next training level and the testing procedure. Conversely, if the cumulative number of successful trials was performed at the chance level in the binomial test, the dog was not admitted to the following step. Video supplementary material including trials from training levels 1, 2, and 3 are given in Online Resource 2.

In the test phase (TEST, Fig. 1C), the experimental setup remained consistent with T3. Dogs were presented with transposed sequences. The order of presentation of these sequences was chosen according to the criterion of maximum distance from the training sequences, starting with the octave equivalent transpositions. Therefore, the dogs were first tested with transpositions of 24- and 12-notes sequences, according to the octave equivalence. Subsequently, 18- and 6-notes transpositions were used, which consisted of different notes compared to the training sequences (Table 1). Video supplementary material including trials from the test phase are provided in Online Resource 3.

### Statistics

As null responses were infrequent and therefore not considered, statistical analyses were performed using a one-sample binomial test, encompassing all the available trials within each training level and the subsequent test phase. Furthermore, the binomial test was applied to the trials of each session separately. Finally, this test was also employed to collectively assess the first 8 responses to the 8 transposed sequences used in the relative sessions. To provide an overview of performance, scores were computed by dividing the number of correct responses by the total number of trials in each session, training level and test. Therefore, the scores ranged from 0 to 1, with higher scores indicating a greater number of correct responses. Statistical analysis was performed using IBM SPSS Statistic version 26 (IBM Corp., Armonk, NY, USA).

## Results

Due to various reasons, some owners were unable to adhere to the study timetable, resulting in the withdrawal of two dogs in T1, and two dogs in T2. Furthermore, ten dogs, including all three dogs that underwent the “*long-training*”, were unable to successfully master the required skill in T3. Consequently, these dogs were excluded from the testing procedure. The performance of each dog, outlining the results of the dogs in the “*short-training*” and the “*long-training*” groups separately, are given in Online Resource 4. The results related to the two remaining dogs that successfully met the criteria and were included in the test phase of the study are described in the following section.

**Fig. 2:**
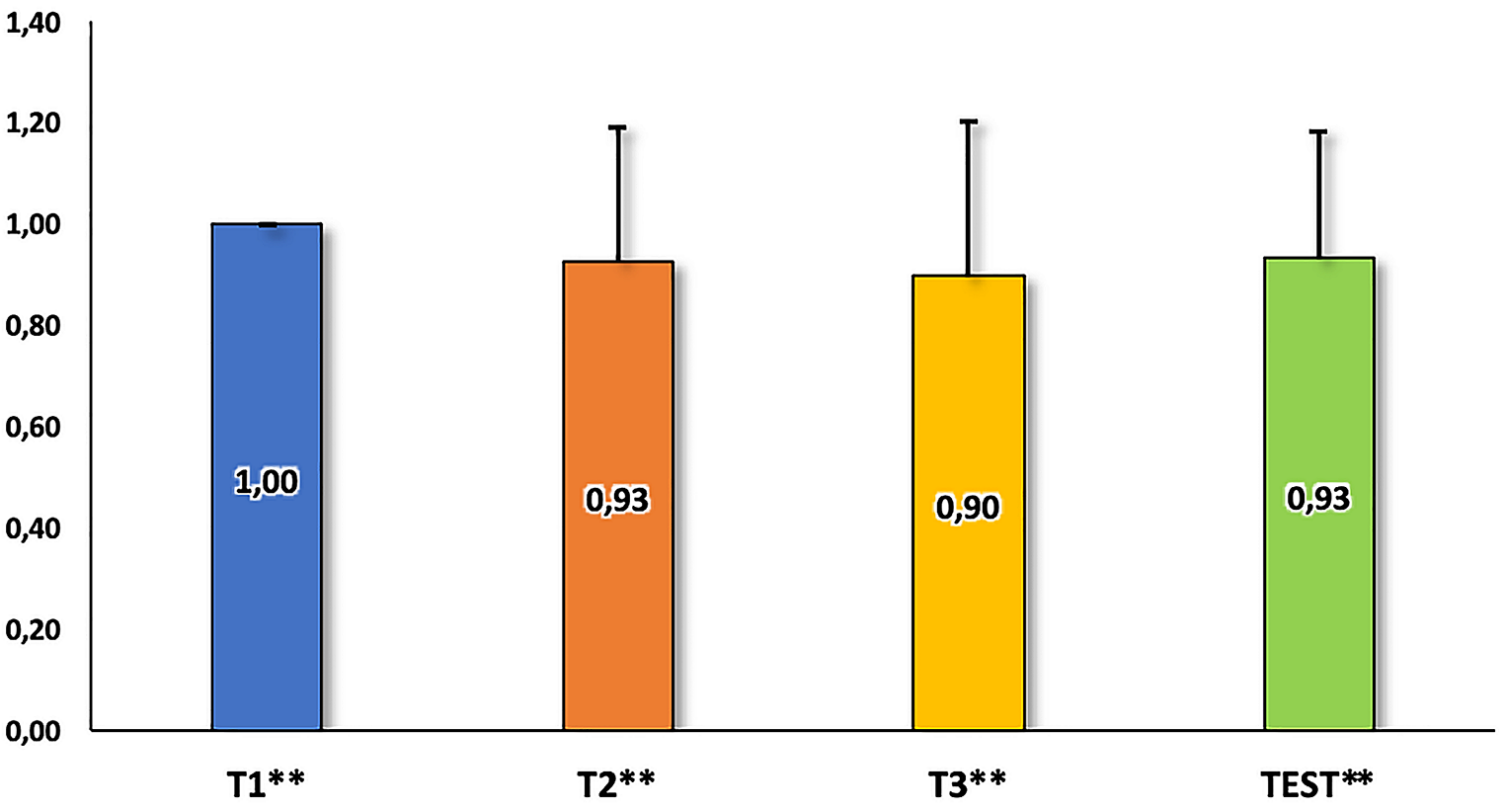
Performance of the dog GR_01_21 in the training levels and the test. The values in the bars represent the frequency of correct responses. All performances were above the chance level according to the binomial test. **p < 0.001

**Fig. 3:**
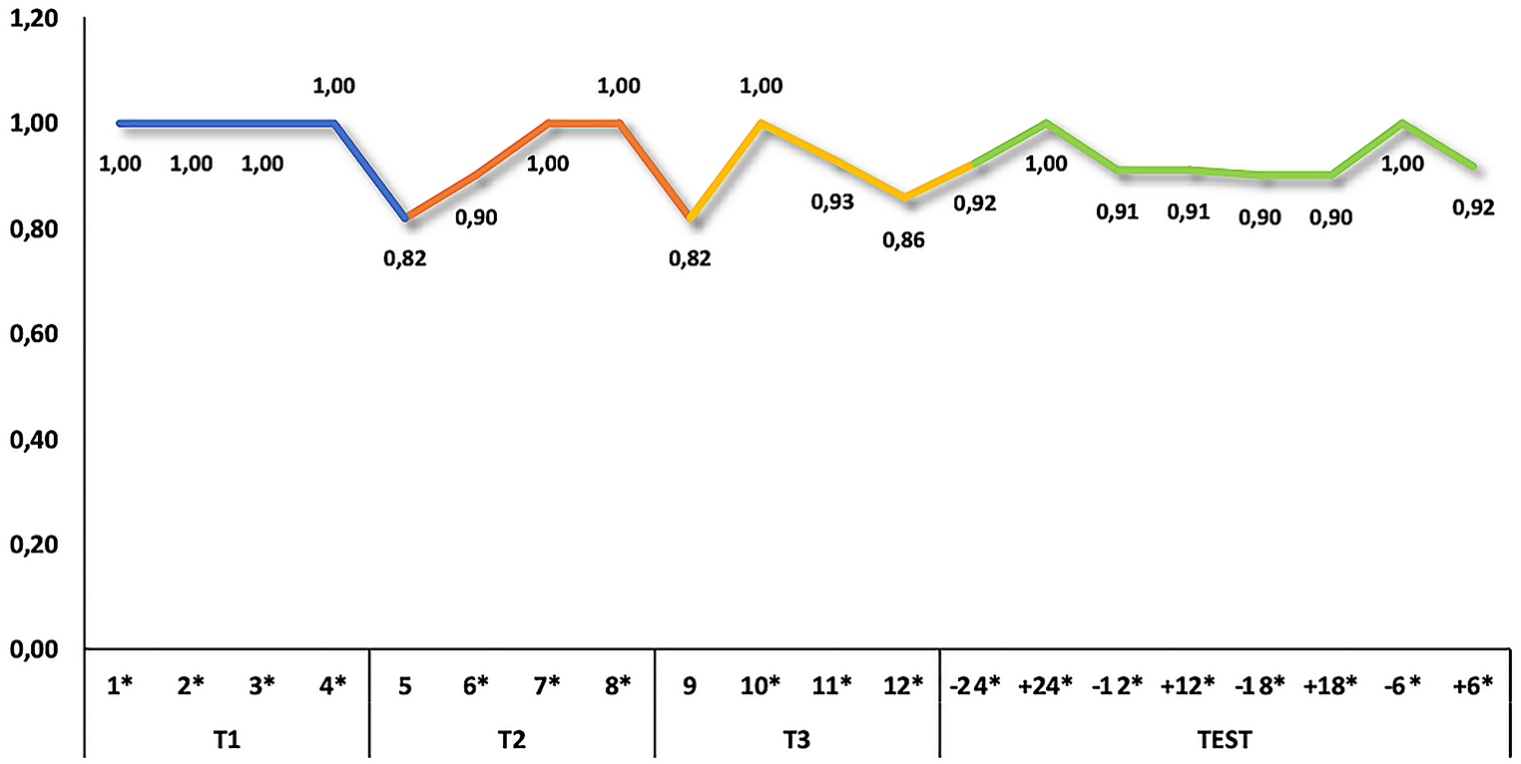
Performance of the dog GR_01_21 in single sessions. According to the binomial test, all single sessions were above chance level, except for trials 5 and 9 which were statistical tendencies (*p* = 0.065). *p < 0.05

***Dog GR_01_21*** (Botch) (Figs. 2 and 3): the dog exhibited careful behavior, demonstrating advanced education and exceptional communication skills with its owner since the beginning of the training. After each trial, the dog autonomously returned to the mat upon command, displaying a high level of self-control. Overall, the dog consistently waited for the verbal command before starting towards the target, displaying calm behavior throughout the entire procedure. There were a few instances where the dog prematurely left the position before hearing the complete sequence of 4 tones, resulting in only 3 null responses throughout the training and testing phase.

In T1, the dog performed flawlessly in all 4 sessions without making any mistakes. The owner did not need to use gestures, as the dog quickly understood that the side of the bowl was the target location. The dog’s performance in this training level was significantly above chance, both in terms of the total number of correct trials (*p* < 0.001) and in each session (*p* < 0.05 in all sessions).

During T2, the owner gradually phased out the use of gestures after the first session, yet the dog still performed above chance in terms of the number of correct responses (*p* < 0.001). In 3 out of the 4 sessions, the dog made a few incorrect trials, but the number of correct trials in three sessions remained above chance (*p* < 0.05), with one session showing a tendency towards significance (*p* = 0.065).

Similar performance was observed in T3, with the overall number of correct trials significantly above chance (*p* < 0.001). Additionally, in 3 sessions, the number of correct trials was above chance (*p* < 0.05), and 1 session showed a tendency towards significance (*p* = 0.065). Consequently, the dog was deemed suitable for the testing procedure.

The test results indicated that the correct responses to the transposed sequences were significantly above chance level, considering all correct trials (*p* < 0.001), as well as those in each session (*p* < 0.05 in all sessions), achieving remarkably high scores. Analysing the responses to first presentation only of each of the transposed sequences revealed that the dog made no errors, which is highly unlikely to occur by chance (*p* = 0.008).

**Fig. 4:**
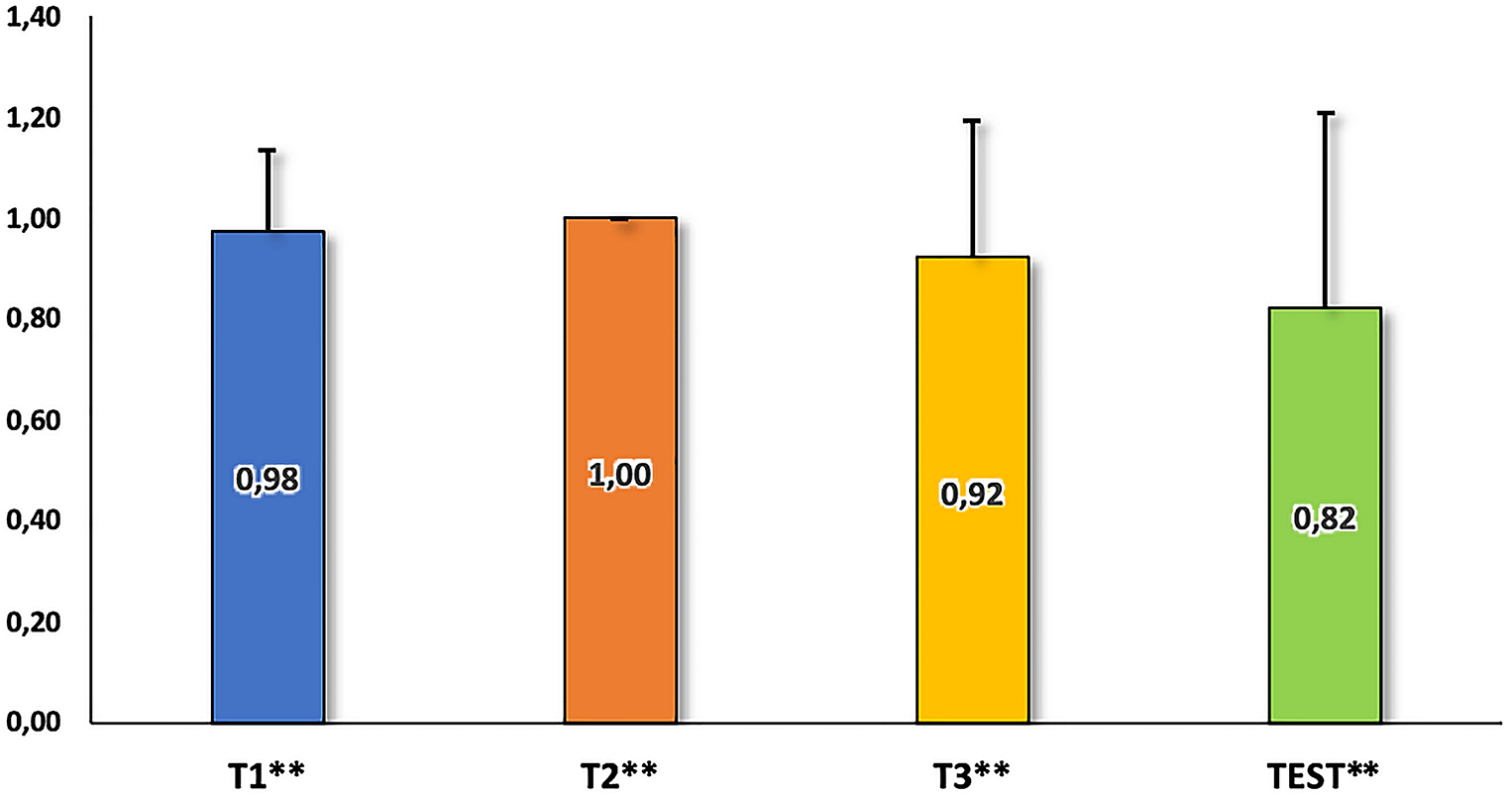
Performance of the dog LHC_01_21 in the training levels and the test. The values in the bars represent the frequency of correct responses. All performances were above the chance level according to the binomial test. **p < 0.001

**Fig. 5:**
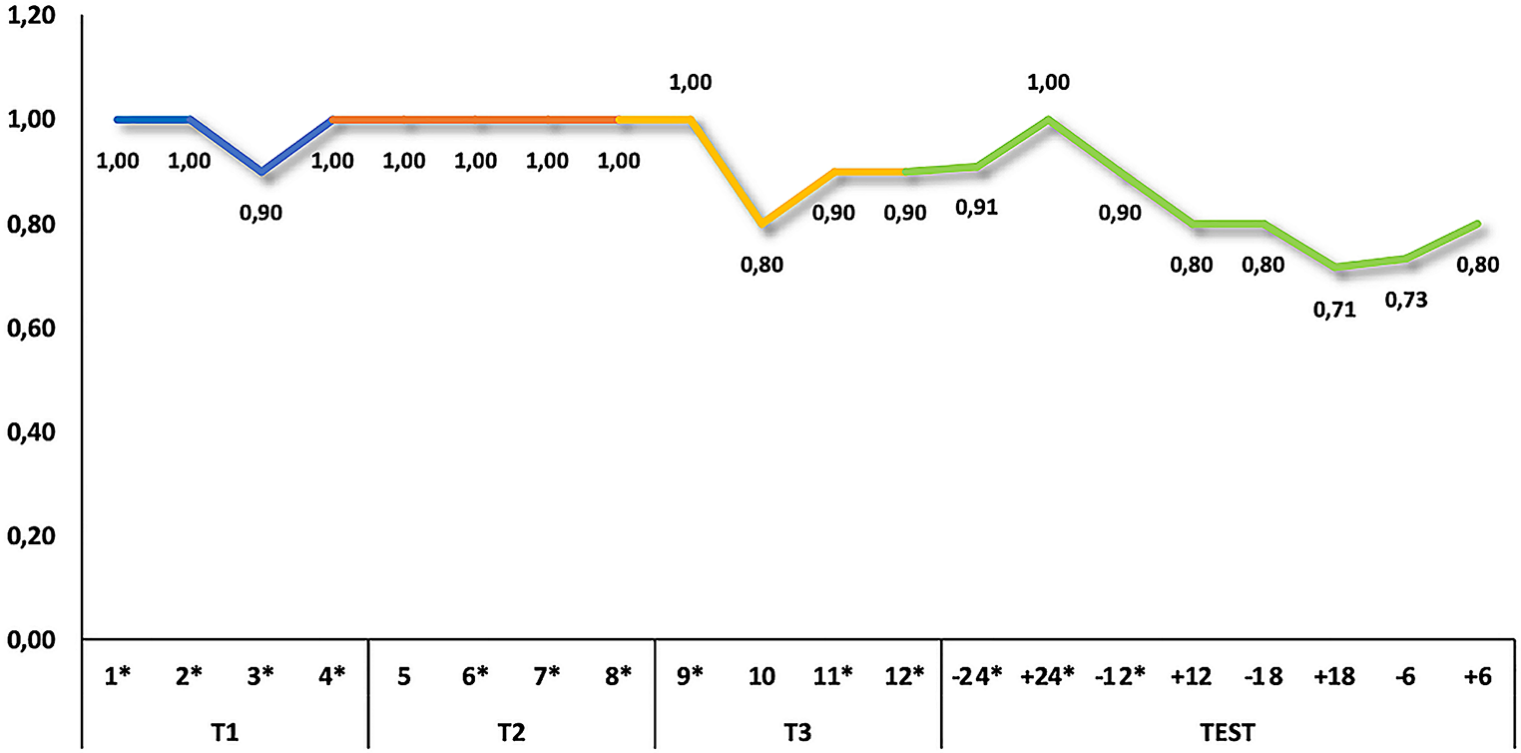
Performance of the dog LHC_01_21 in single sessions. According to the binomial test, most of the sessions were above the chance level. *p < 0.05

***Dog LHC_01_21*** (Maffolo) (Figs. 4 and 5): the dog was highly active and promptly responded to the stimuli. However, due to its extremely proactive temperament, it often exhibited anticipatory movements, leaving its position before the sequence of tones was played. As a result, 12 trials had to be nullified. Despite this, the dog quickly learned to start from the designated position and reach the side bowl after the sound sequence ended and without requiring a verbal prompt from the owner.

In T1, the dog made only a mistake in the third session. Overall, its performance was significantly above chance in terms of the number of correct trials in this training level (*p* < 0.001) and in each session (*p* < 0.05 in all sessions).

In T2, the owner eliminated the use of gestures in most of the trials after the first session, and the dog performed flawlessly, making no mistakes. The binomial test indicated that the overall performance, considering all correct trials in T2, was significantly above chance level (*p* < 0.001), as well as the correct trials of each session (*p* < 0.05 in all sessions).

In T3, the cumulative trials yielded results above chance regarding the number of correct responses (*p* < 0.001), as well as in 3 sessions (*p* < 0.05). Based on these results, the dog was admitted to the testing procedure.

During the test phase, the correct responses were overall above chance level (*p* < 0.001). When considering the transpositions that respected the octave transposed test sequences, the dog performed above chance in the correct trials in 3 sessions (*p* < 0.05). However, when the transpositions did not respect the octave transposed test sequences, although the dog achieved a high score, its performance was at chance level.

Analysing the responses to first presentation only of each of the transposed sequences revealed that the dog made no errors (*p* = 0.008).

## Discussion

The present study reveals that while most dogs exhibited performance above chance levels when their owners were visible, their accuracy significantly decreased when their owners were out of sight. This implies that dogs heavily depended on visual cues involuntarily given by their owners and were unable to switch to auditory cues when their owners were out of sight. This phenomenon could not be attributed to the “*short*” duration of the training, since even dogs that underwent “*long-training*” periods were unable to master the task. Consequently, it suggests that dogs may rely more on visual cues than auditory stimuli in certain contexts. On the other hand, existing research (D’Aniello et al. 2016; Scandurra et al. 2017, 2018) has already established that dogs tend to prioritize visual cues over acoustic signals, which can explain the low success in shifting to the sequence of musical tones observed in our study. However, despite the general challenge faced by dogs in acquiring the task, even for those long-trained, it is noteworthy that 2 dogs in our study were able to learn the task in a remarkably short (12 sessions with a minimum of 10 trials per session) training period. This suggests that these dogs were able to memorize and attend to relative pitch.

We wanted to test whether dogs use relative pitch in a familiar context to promote comfort and attention to the task at hand. However, as a result we had less control of the testing situation. Therefore, we asked for video recordings of the sessions so that we could check for cues that may have been used by the dogs to solve the task instead of the test stimuli.

Despite our efforts to minimize potential harmonic effects by generating sinusoidal sounds, the use of mobile devices for sound reproduction likely introduced unintended harmonic distortions. While in our study it’s uncertain whether dogs have the ability to perceive harmonics as distinct sounds, it’s not entirely dismissible. Critically, harmonic distortions may have presented the dogs with the same frequency information as octave transpositions. If the dogs had memorized the training stimuli, they may have used the frequency information in these distortions to solve the task. However, while this may be an explanation for how one of the two successful dogs solved the task, the other dog solved the task even for other kinds of stimulus transpositions, which suggests that it is possible for dogs to generalize relative pitch patterns.

The ability to encode relative pitch and perceive melodic invariance across pitch transpositions is a sophisticated ability (Plantinga and Trainor 2005). For example, zebra finches trained to discriminate between two different songs could only recognize those songs within a limited range of transpositions (Nagel et al. 2010). However, with appropriate training, some birds (Braaten et al. 1990; Cynx 1995; MacDougall-Shackleton and Hulse 1996) and mammal species (Izumi 2001; Yin et al. 2010) can be taught to discern the relative pitch relationships of sound sequences over a wider range. The previously cited old studies conducted in dogs (Kalischer 1907; Selionyi 1907) showed that while recognizing different sounds and notes is an easy task, it becomes more challenging for dogs to distinguish notes with different pitches but the same timbre (Shepherd 1919). Notes and sounds are all related to the perception of auditory stimuli but recognizing the pitch of notes when transposed by an octave may require different and more cognitive demands. Therefore, it is not surprising that in the current study, only 2 out of 16 dogs were able to accurately differentiate between the two sound sequences and their transpositions. It is worth noting that these two dogs demonstrated a remarkable ability to discriminate transpositions and, notably, without any prior training.

Recent research has focused on identifying genetic factors that contribute to human musical abilities. Twin studies and familial clustering have demonstrated a high heritability for certain musical skills (Drayna et al. 2001). Specific chromosomal regions have been associated with musical aptitude and individual genes have been found to impact creativity and musical abilities (Gingras et al. 2015). Our results support the perspective that there exists considerable individual variation in dogs’ capacity to learn and differentiate relative pitch information. We also know that dogs show high individual variation in other sensory capacities like smell abilities, and high genetic diversity within and among breeds (Kokocińska-Kusiak et al. 2021). Indeed, variability in musical abilities is observed in humans as well. Relative pitch perception is typically an innate ability, or at the very least, quickly acquired, in most individuals and a fundamental skill for musicians (McDermott and Hauser 2005). However, some individuals, such as those with amusia, may lack this ability (Cousineau et al. 2012). Therefore, musicality is a personal characteristic that can be present in certain individuals, whether they are humans, dogs, or potentially other non-human species. This variability may help explain why studies exploring musical abilities in animals often yield limited results. It is possible that these studies did not successfully select experimental subjects with significant musical skills. On the other hand, some studies have emphasized that certain dogs may exhibit exceptional giftedness, laying the groundwork for establishing dogs as a model species for studying talent (Fugazza et al. 2021). Thus, the key message from the current study is that future research on musicality may yield more valuable insights by focusing on individual subjects rather than groups. This shift is crucial because musical abilities may potentially be present in all species, albeit occurring rarely within specific individuals. Additionally, developing protocols to mitigate potential acoustic Clever Hans effects (e.g., prosodic information in the owner’s calls) would be crucial in ensuring the validity of findings.

The potential presence of musical abilities in certain dogs poses intriguing questions about the origin and nature of musicality in canines. One possible explanation for this phenomenon is that musicality is a by-product of natural or artificial selection. However, it is important to note that wolves and other members of the Canidae family have not been extensively studied regarding their musical abilities. Wolves, for example, engage in group howls that involve sustained vocalizations and pitch modulation, which share similarities with human singing (Theberge and Falls 1967). Each individual within the pack exhibits its own pitch and tone variations (Tooze et al. 1990). Moreover, wolf howl frequencies differ in consistent ways, implying a form of modulation in response to the calls of others (Filibeck et al. 1982). This vocal modulation could be seen as an indication of a potential primordial form of musicality in wolves. Vocal accommodation is a phenomenon where animals modify their vocalizations in response to the vocal signals of others, playing a role in communication and social dynamics within animal groups (Janik and Slater 2000). However, while vocal accommodation demonstrates a sensitivity to auditory input, it does not necessarily imply a direct link to musicality in wolves. Therefore, it remains uncertain whether the musicality observed in dogs already existed in the ancestral wolf populations.

While there is certainly a need to enrich our knowledge about the evolution and selectivity of these abilities in species not traditionally associated with musical communication, this study adds a piece to the “harmony” of our understanding of the biology of the universal language of music.

## Electronic supplementary material

Below is the link to the electronic supplementary material.

Supplementary Material 1

Supplementary Material 2

Supplementary Material 3

Supplementary Material 4

## Data Availability

All tables and graphical data obtained during this study are included in this published article. The datasets generated and/ or analyzed during the current study are available from the correspond ing author on request.
