# Correction: In “Tone” with dogs: exploring canine musicality

**DOI:** 10.1007/s10071-025-01994-7

**Published:** 2025-07-25

**Authors:** Claudia Pinelli, Anna Scandurra, Cristina Giacoma, Alfredo Di Lucrezia, Biagio D’Aniello

**Affiliations:** 1 Department of Environmental, Biological and Pharmaceutical Sciences & Technologies, University of Campania “Luigi Vanvitelli”, Caserta, 81100 Italy; 2 Department of Biology, University of Naples Federico II, Naples, 80126 Italy; 3 Department of Life Sciences and System Biology, University of Torino, Torino, 10123 Italy

**Correction: Animal Cognition (2024) 27:38**

10.1007/s10071-024-01875-5

In this article the ‘Conflict of Interest’ statement was missing and should have been “Biagio D’Aniello is an editor for the journal and has not taken part in the manuscript’s peer review process.” The original article has been corrected.

