# Supplementary material for: In “Tone” with dogs: exploring canine musicality
Source: Anim Cogn. 2024 May 16;27(1):38. doi: 10.1007/s10071-024-01875-5 (PMC11096221; doi:10.1007/s10071-024-01875-5)
Supplement: Supplementary file 1 — Supplementary Material 1 [file 10071_2024_1875_MOESM1_ESM.pdf]

In "Tone" With Dogs: Exploring Canine Musicality

Claudia **Pinelli**[1], Anna **Scandurra**[2], Cristina **Giacoma**[3], Alfredo **Di Lucrezia**[2], Biagio **D'Aniello**[2]*

[1] Department of Environmental, Biological and Pharmaceutical Sciences & Technologies, University of Campania "Luigi Vanvitelli", 81100, Caserta, Italy.
[2] Department of Biology, University of Naples Federico II, 80126, Naples, Italy.
[3] Department of Life Sciences and System Biology, University of Torino, 10123, Torino, Italy

	ID	Breed	Age (yrs.)	Sex	Weight (Kg)	Previous training	Owner experience	N° trials			
								T1	T2	T3	TEST
1	B_01_21	Beagle	3	F	15	Basic	Experimenter	41	49	46	NA
2	BC_01_21	Border Collie	2	F	18	Basic	Experimenter	40	344	47	NA
3	D_01_21	Deerhound	8	M	18	Basic	Dog trainer	40	40	NA	NA
4	GR_01_21	Golden retriever	3	M	35	Water rescue dog	Dog trainer	41	41	49	90
5	GR_02_22	Golden Retriever	6	M	35	Water rescue dog	Dog trainer	40	48	52	NA
6	GR_03_22	Golden Retriever	3	F	30	Water rescue dog	Dog trainer	49	48	49	NA
7	GS_01_21	German shepherd	7	M	30	Basic	Experimenter	42	266	44	NA
8	L_01_21	Labrador	7	F	40	Water rescue dog	Dog tainer/Experimenter	51	333	42	NA
9	L_01_22	Labrador	4	F	36	Water rescue dog	Dog trainer	51	49	45	NA
10	L_02_22	Labrador	4	M	33	Water rescue dog	Dog trainer	40	42	NA	NA
11	LC_01_22	Labrador chocolate	4	F	27	Water rescue dog	Dog trainer	47	NA	NA	NA
12	LHC_01_21	Long haired chihuahua	5	M	5	Basic	Dog trainer	40	40	39	90
13	LR_01_22	Labrador retriever	10	F	31	Water rescue dog	Dog trainer	51	48	51	NA
14	LR_02_22	Labrador retriever	3	F	29	Water rescue dog	Dog trainer	48	NA	NA	NA
15	M_01_22	Mixed	3	F	15	Basic	Dog trainer	47	48	48	NA
16	M_02_22	Mixed	10	F	6	Basic	Dog trainer	46	47	46	NA
