# Supplementary material for: In “Tone” with dogs: exploring canine musicality
Source: Anim Cogn. 2024 May 16;27(1):38. doi: 10.1007/s10071-024-01875-5 (PMC11096221; doi:10.1007/s10071-024-01875-5)
Supplement: Supplementary file 4 — Supplementary Material 4 [file 10071_2024_1875_MOESM4_ESM.pdf]

In "Tone" With Dogs: Exploring Canine Musicality

Claudia **Pinelli**[1], Anna **Scandurra**[2], Cristina **Giacoma**[3], Alfredo **Di Lucrezia**[2], Biagio **D'Aniello**[2]*

[1] Department of Environmental, Biological and Pharmaceutical Sciences & Technologies, University of Campania "Luigi Vanvitelli", 81100, Caserta, Italy.
[2] Department of Biology, University of Naples Federico II, 80126, Naples, Italy.
[3] Department of Life Sciences and System Biology, University of Torino, 10123, Torino, Italy

SHORT-TRAINING

Dog LR_01_22 (Fig. 1, 2): the dog exhibited high levels of excitement during the training sessions. However, it remained highly attentive to its owner and responded well to the signals. Notably, the dog did not make any null responses throughout the entire training procedure, indicating its careful participation to the procedure. The dog quickly learned to leave the position after the sequence was played without needing a verbal invitation from the owner.

During T1, the dog made some mistakes in the third session, resulting in a performance at chance level. However, the number of correct trials in the other three sessions was above chance ($p<0.05$). Overall, all the correct trials in T1 were significantly above chance ($p<0.001$).

In T2, the dog's performance was at chance level in 3 out of 4 sessions. However, when considering all the correct trials at this training level, the overall performance was significantly above chance ($p<0.05$).

In T3, both the cumulative correct trials and those in each single session fell at chance level.

Based on these results, the dog was not admitted to the testing procedure, as it did not demonstrate the required proficiency.

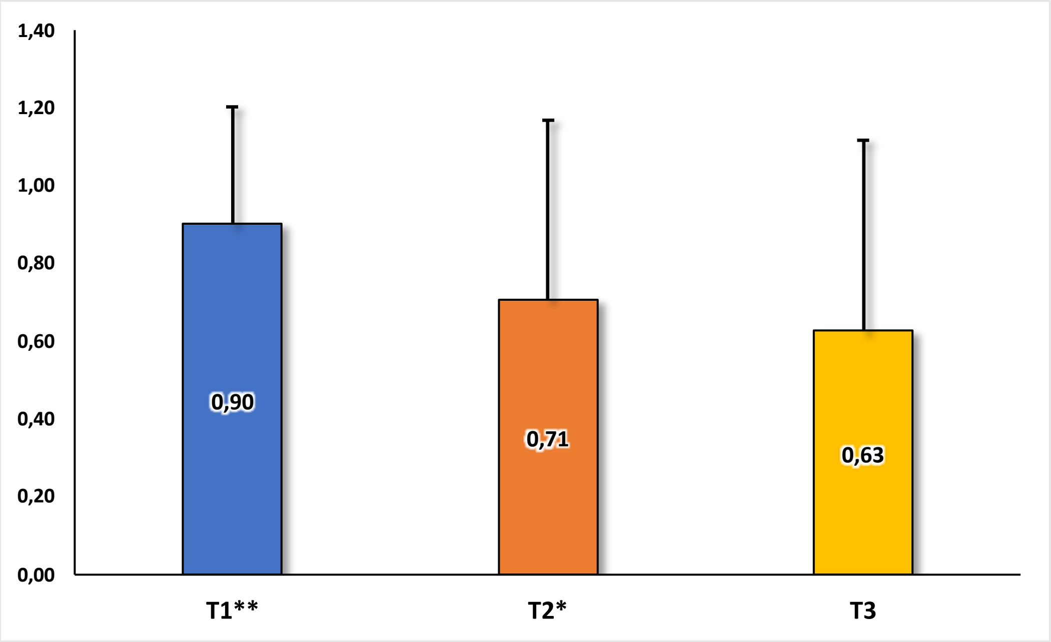

Fig. 1 Performance of LR_01_22 in the training levels. Training 1 and 2 were above chance level according to the binomial test. *p<0.05; **p<0.001

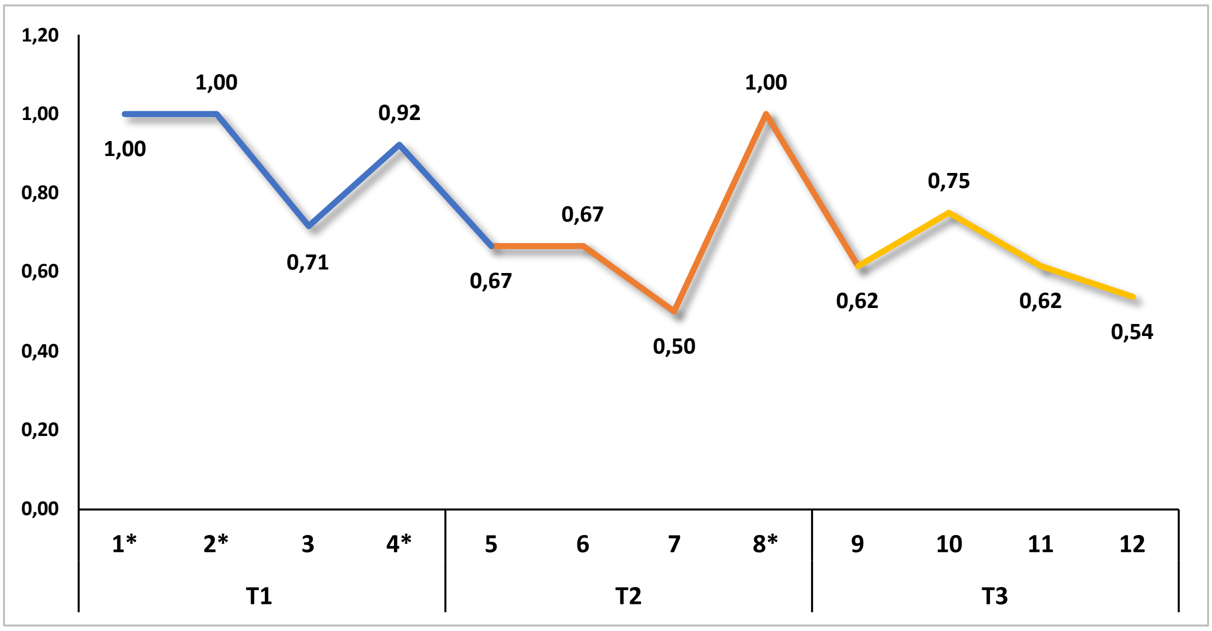

Fig. 2 Performance of LR_01_22 in single sessions. According to the binomial test, most of the sessions were below chance level. *p<0.05

Dog L_01_22 (Fig. 3, 4): the dog displayed a high level of focus on its owner and demonstrated careful attention to signals throughout the training sessions. As a result of effective training, the dog learned to initiate actions after hearing the sound sequence without the need to rely on verbal prompts. However, during 6 trials, the dog exhibited anticipatory responses, which were considered null.

In T1, despite some mistakes, the dog consistently performed the correct responses in individual sessions above chance levels ($p<0.05$). Overall, the total number of correct responses in this training level was significantly above chance expectations ($p<0.001$).

Moving on to T2, the dog achieved chance-level performance in 2 sessions, while in the other 2 sessions, the number of correct responses exceeded chance levels ($p<0.05$). The overall number of correct responses in this training level was also significantly above chance ($p<0.001$).

However, during T3, both the cumulative trials and those in the individual sessions resulted in chance-level performance.

Consequently, based on these findings, the dog did not meet the required criteria for admission into the testing procedure.

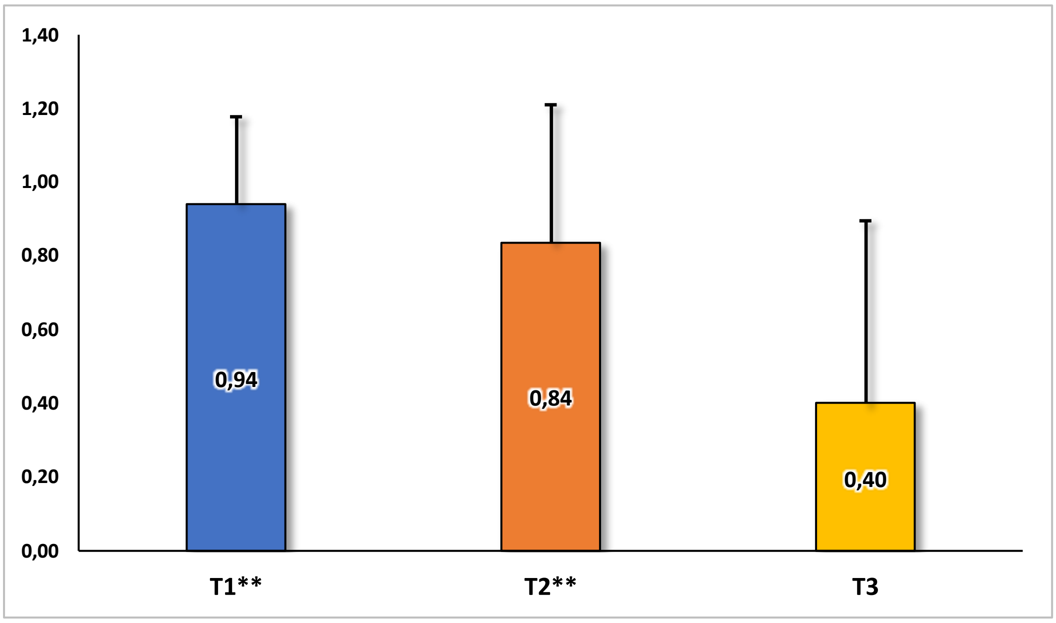

Fig. 3 Performance of L_01_22 in the training levels. T1 and T2 were above chance level according to the binomial test. **$p<0.001$

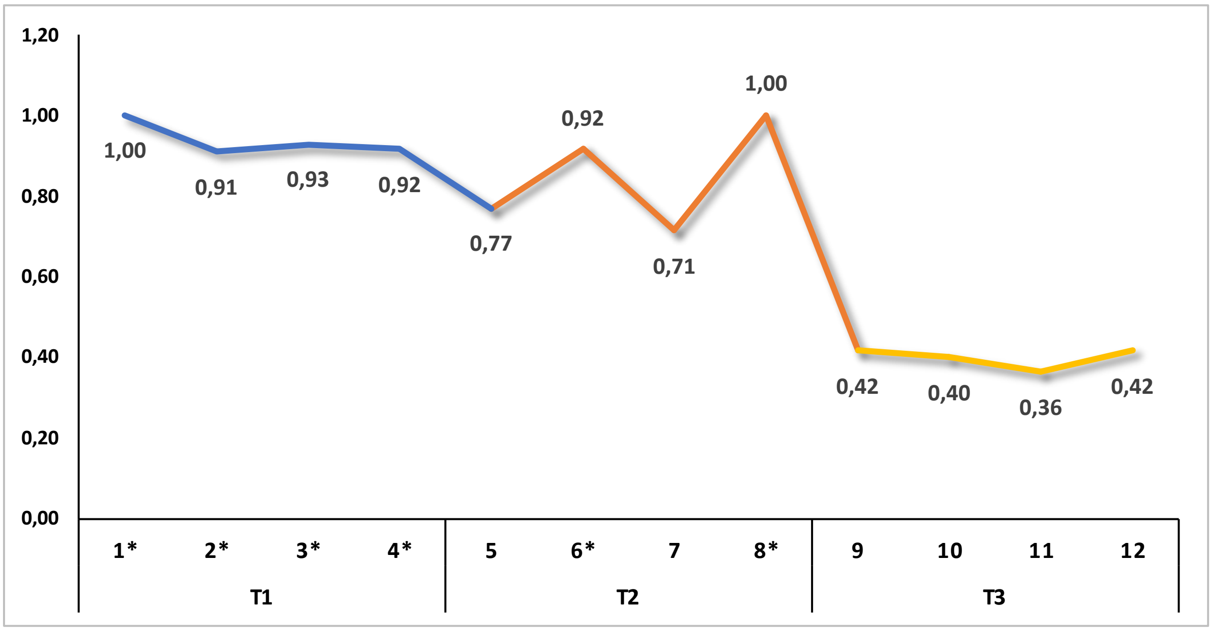

Fig. 4 Performance of L_01_22 in single sessions. According to the binomial test, half of the sessions were above chance level. *p<0.05

Dog GR_02_22 (Fig. 5, 6): the dog exhibited calm and controlled behavior, moving slowly but attentively towards the target. Following the playback of the sound sequences, the dog required verbal encouragement from the owner to leave its position and approach the bowl. Remarkably, the dog did not make any null trials throughout the entire training process.

During T1, the dog's performance was flawless, with no mistakes observed in any of the sessions . Moreover, the cumulative number of correct trials in this training level was significantly above chance ($p<0.001$).

A good performance was also observed in T2, with 3 mistakes occurring in the first session, which was at chance level. However, there were no errors in the remaining 3 sessions ($p<0.05$). Overall, the number of correct trials in this training level was significantly above chance ($p<0.001$).

In T3, both the cumulative correct trials and those in each individual session resulted in chance-level performance.

As a result, based on these findings, the dog did not meet the criteria for admission to the testing procedure.

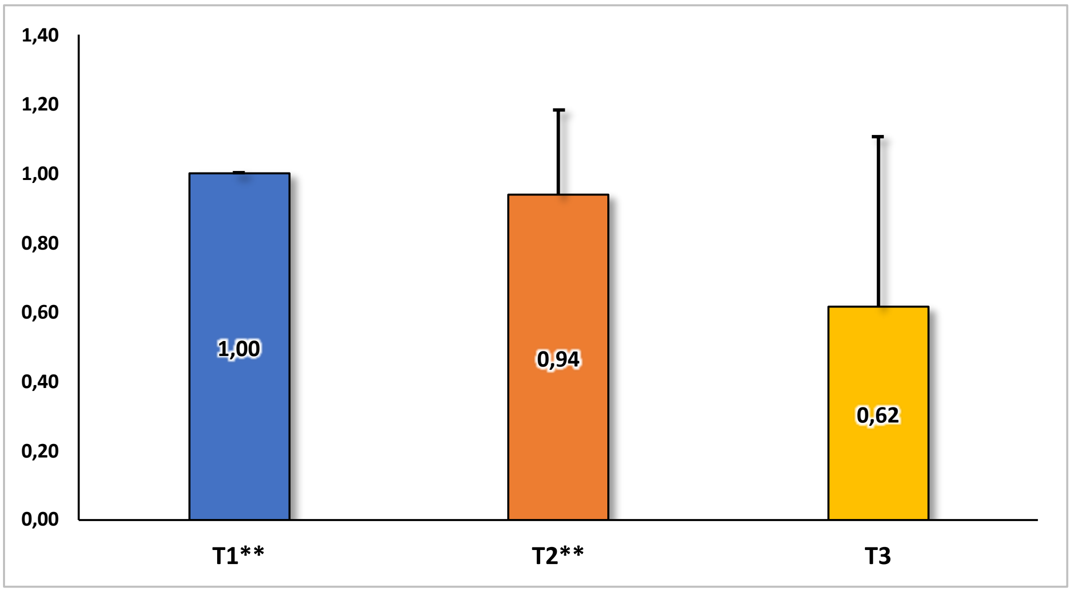

Fig. 5 Performance of GR_02_22 in the training levels. T1 and 2 were above chance level according to the binomial test. **p<0.001

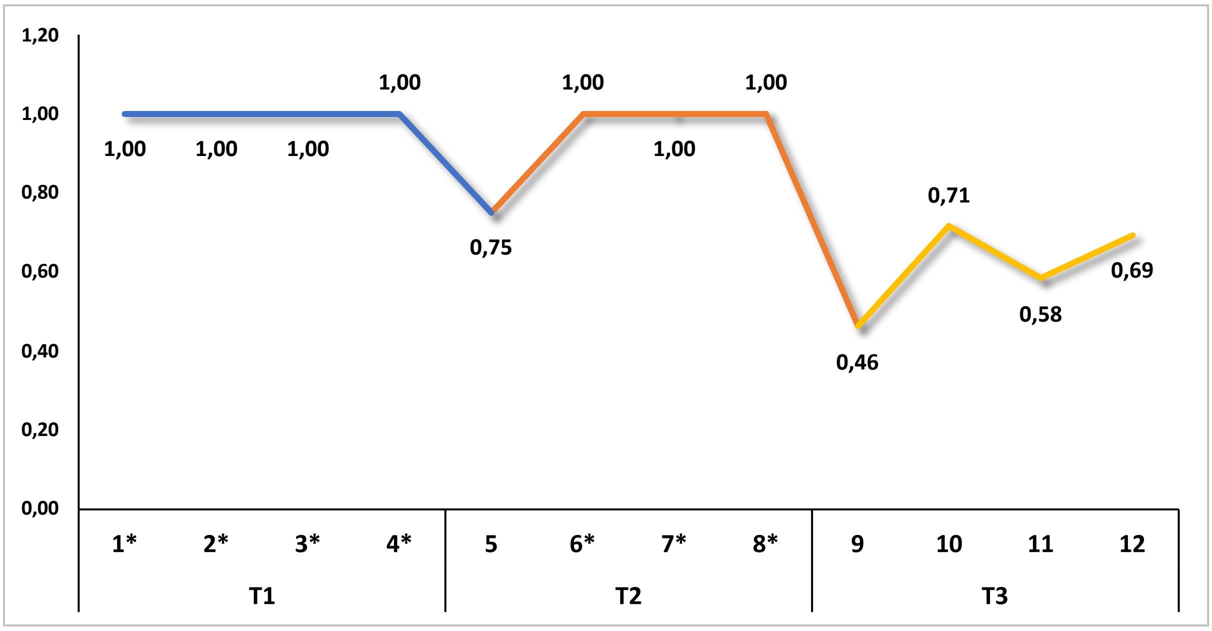

Fig. 6 Performance of GR_02_22 in single sessions. According to the binomial test, most of the sessions were above chance level. *p<0.05

Dog GR_03_22 (Fig. 7, 8): the dog exhibited a dynamic yet cautious behavior towards its owner, remaining attentive and responsive to signals throughout the training sessions. After the sound sequence was played, the dog required verbal prompting before leaving its position. The dog displayed anticipatory behavior in 2 trials that had to be nullified.

During T1, the dog made some mistakes. However, the number of correct trials in each individual session was significantly above chance ($p<0.05$), as well as that in the overall performance in this training level ($p<0.001$).

Regrettably, T2 resulted in poor performance, with all sessions and the overall performance falling at chance level.

Similarly, T3 also yielded unsatisfactory results, leading to the decision not to admit the dog to the test.

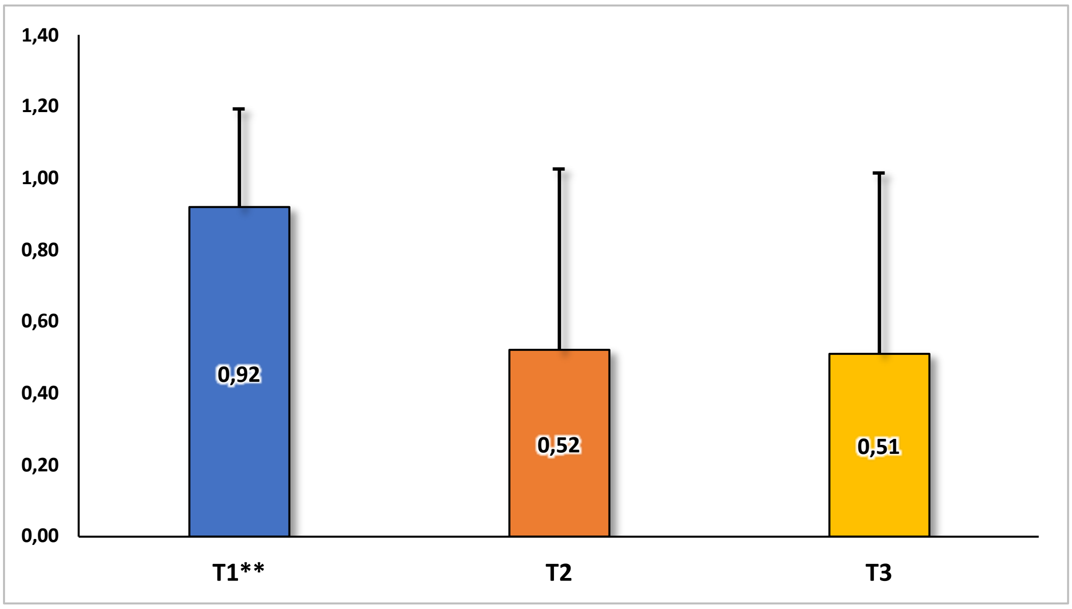

Fig. 7 Performance of GR_03_22 in the training levels. According to the binomial test, only T1 was above chance level. **$p<0.001$

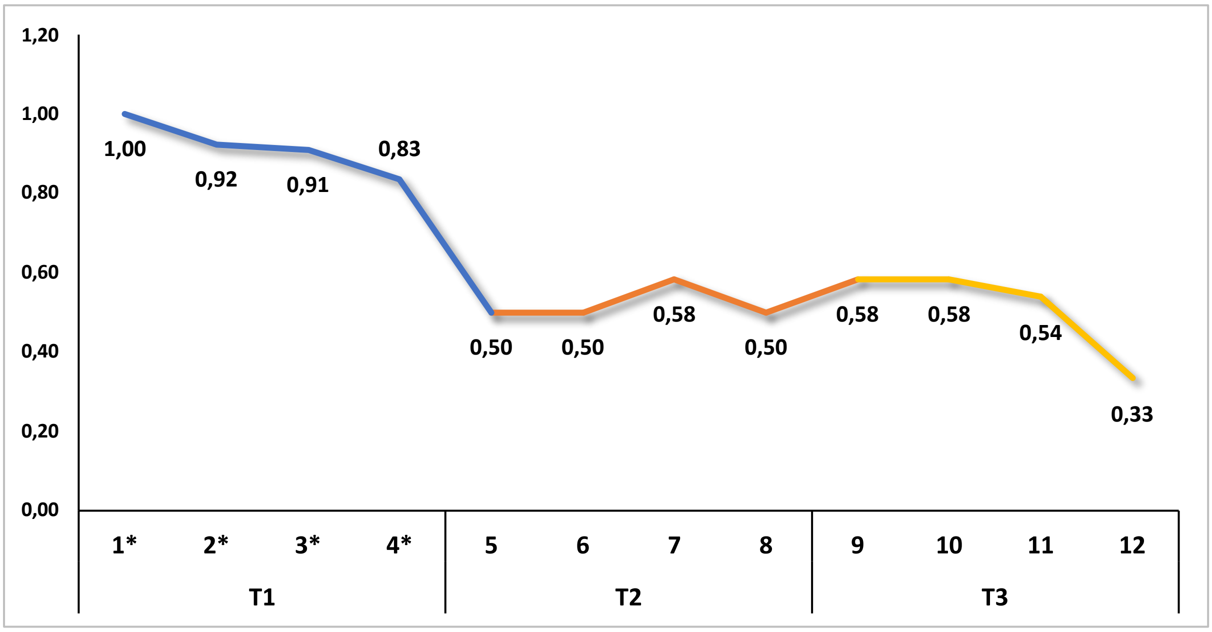

Fig. 8 Performance of GR_03_22 in single sessions. According to the binomial test, most of the sessions were below chance level. *p<0.05

<u>Dog M_01_22</u> (Fig. 9, 10): the dog exhibited a reflective nature, quickly learning to leave its position solely based on the stimulus provided by the sound sequence, with only one null response observed.

During T1, the dog performed flawlessly, without making mistakes. It consistently achieved performance above chance levels, with the number of correct responses in each single session ($p<0.05$) and in the overall training level ($p<0.001$).

However, in both T2 and T3, as well as in each individual session of the two training levels, the number of correct trials was performed at the chance levels. Then the dog was not selected for the test.

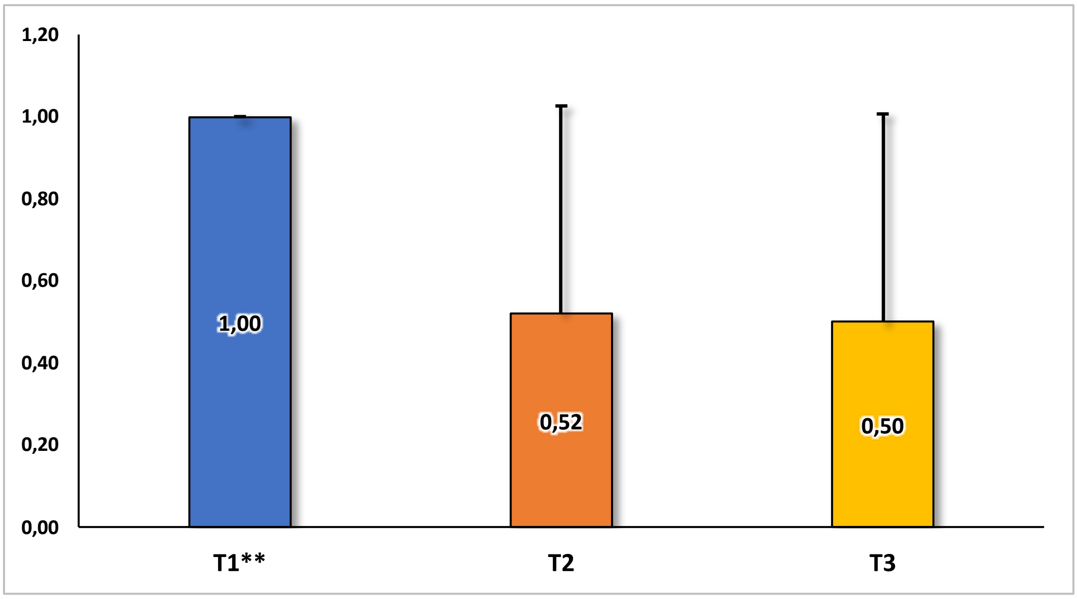

Fig. 9 Performance of M_01_22 in the training levels. According to the binomial test, only T1 was above chance level. **$p<0.001$

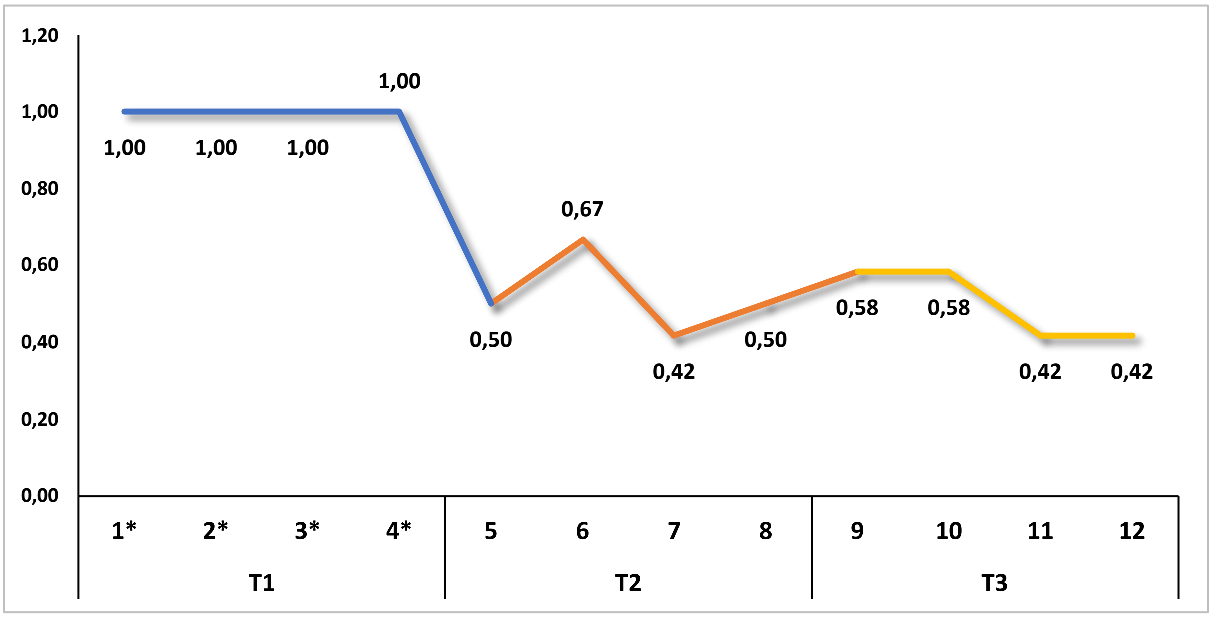

Fig. 10 Performance of M_01_22 in in single sessions. According to the binomial test, most of the sessions were below chance level. *p<0.05

Dog M_02_22 (Fig. 11, 12): the dog displayed high levels of activity and a strong focus on the task. Generally, the owner provided a verbal command after playing the sound sequence, although occasionally the dog initiated the action immediately after the sound sequence. The dog exhibited only 2 null responses throughout the training.

During T1, the dog performed perfectly, without making any mistakes in all sessions. Furthermore, the cumulative number of correct trials in this training level exceeded chance levels ($p<0.001$).

A very commendable overall performance was also observed in T2, with the number of correct trials surpassing chance levels ($p<0.001$). Although the dog made some mistakes, resulting in performance above chance levels in only 2 out of 4 sessions ($p<0.05$), the overall performance in T2 remained notably.

Unfortunately, in T3, all sessions and cumulative trials were performed at chance levels. Consequently, the dog was not admitted to the test.

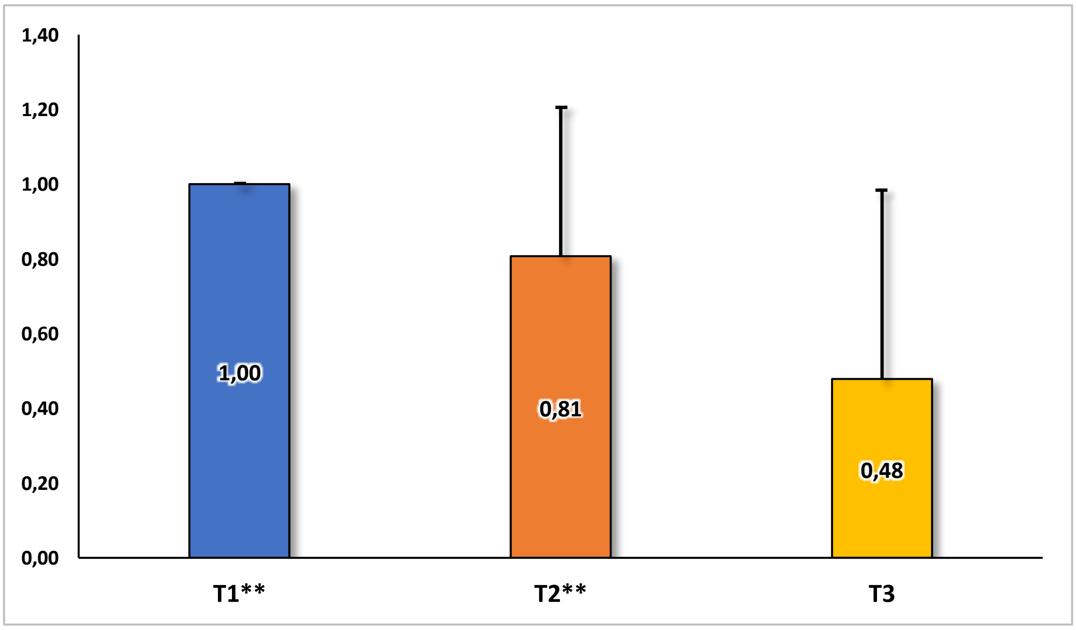

Fig. 11 Performance of M_02_22 in the training levels. According to the binomial test, T1 and T2 were above chance level. **p<0.001

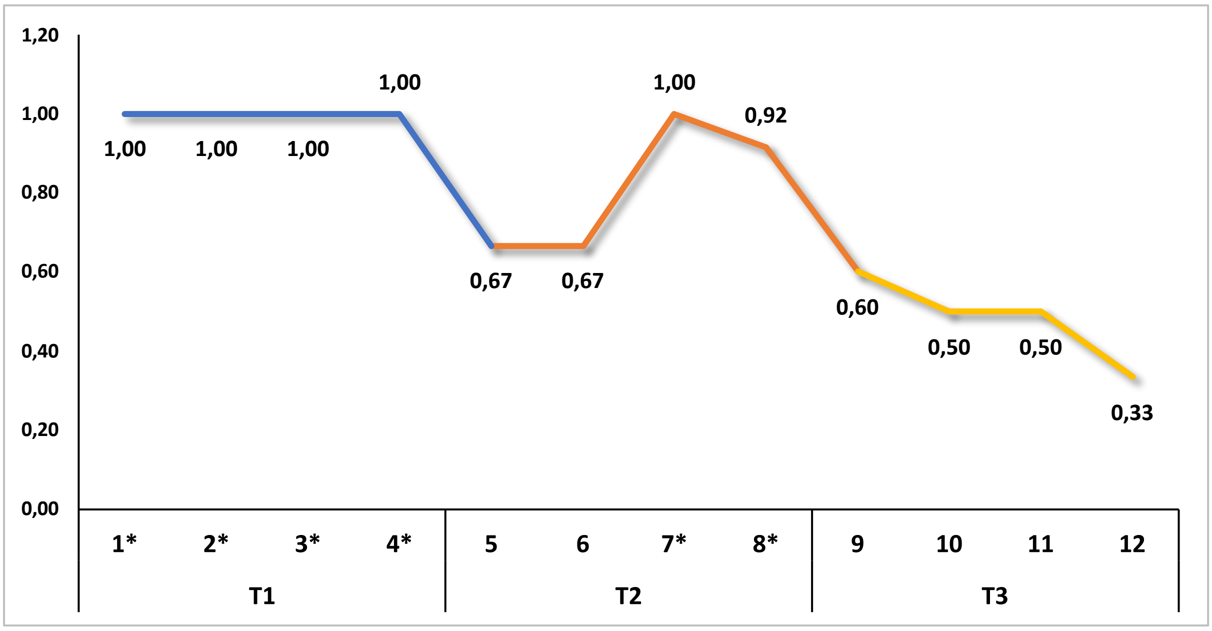

Fig. 12 Performance of M_02_22 in in single sessions. According to the binomial test, half of the sessions were above chance level. *p<0.05

Dog B_01_21 (Fig. 13, 14): The dog displayed a high level of focus and enthusiasm throughout the training sessions, showing great attentiveness to its owner, who also happened to be one of the experimenters. The dog rapidly acquired the ability to react to the sound sequence by immediately leaving its position, without requiring verbal prompting. However, there were instances of anticipatory behavior, which led to the nullification of three trials.

During T1, the dog demonstrated excellent performance, surpassing chance levels in both a global context ($p<0.001$) and in three out of four sessions ($p<0.05$), as indicated by the number of correct trials.

Satisfactory performance was also achieved in T2, with the number of correct trials surpassing chance levels ($p<0.05$) in 2 out of 4 sessions, and in the overall training ($p<0.001$).

Unfortunately, T3 did not meet the required expectations. The dog's performance in both individual sessions and cumulative trials fell within chance levels, leading to the decision not to admit the dog to the test.

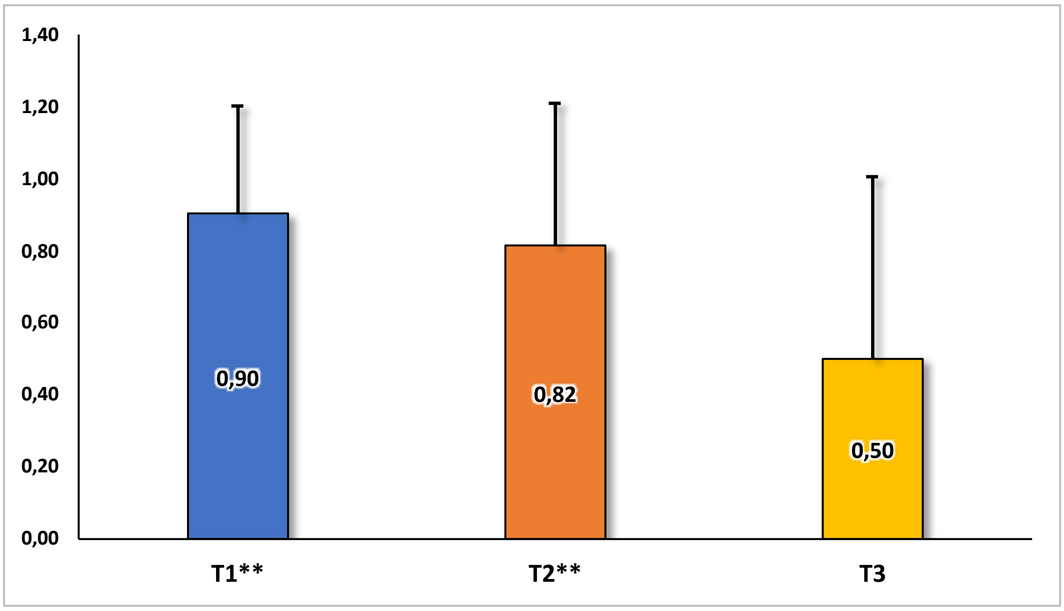

Fig. 13 Performance of B_01_21 in the training levels. According to the binomial test, T1 and T2 were above chance level. **$p<0.001$

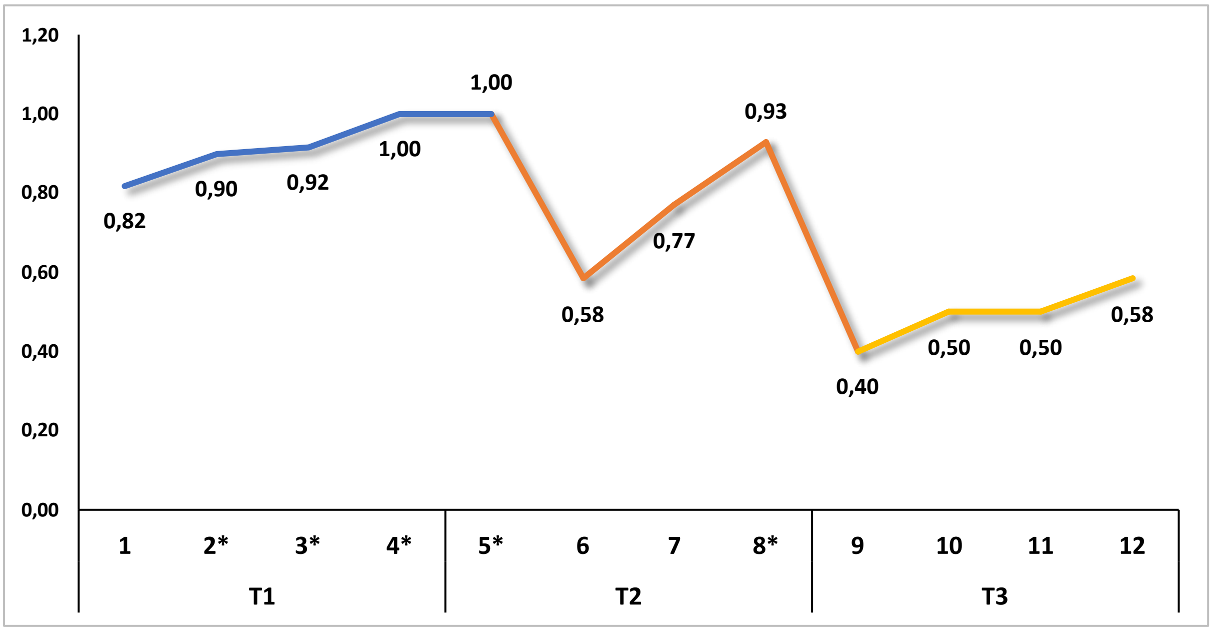

Fig. 14 Performance of B_01_21 in single sessions. According to the binomial test, most of the sessions were above chance level. *p<0.05

Dog L_02_22 (Fig. 15, 16): The dog demonstrated a good level of focus and attentiveness during the training sessions, showing a great communicative relationship with its owner. Occasionally, the dog did not immediately respond to the sound sequence; whereby the owner had to provide a verbal command after playing the sound sequence. Overall, there were no trials to be nullified throughout the whole training process.

In T1, the dog achieved a hight score, performing significantly above chance levels in the overall number of correct responses ($p<0.001$) as well as in each individual session ($p<0.05$).

The same commendable performance was observed in T2, with the number of correct trials significantly exceeding chance levels ($p<0.05$) in 3 out of 4 sessions.

Regrettably, T3 was not conducted as the owner chose not to proceed due to personal engagements.

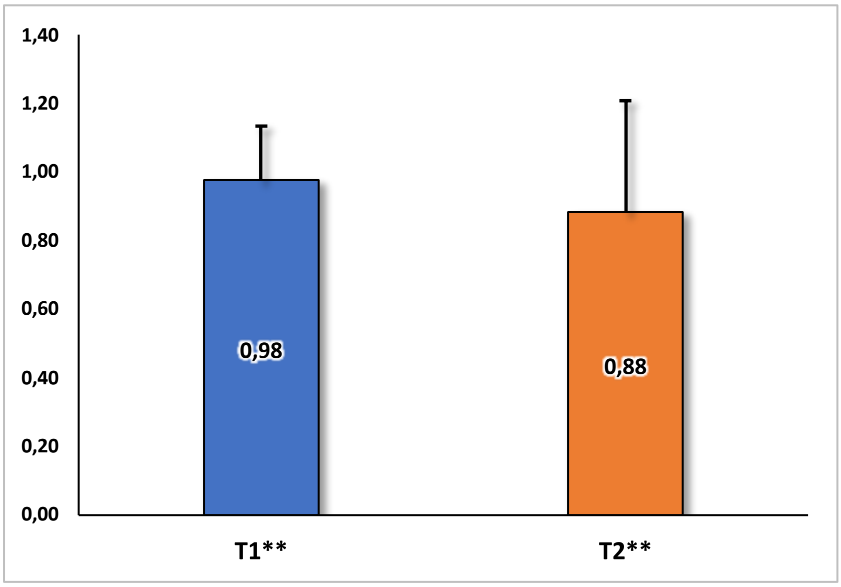

Fig. 15 Performance of L_02_22 in the training levels. According to the binomial test, T1 and T2 were above chance level. **p<0.001

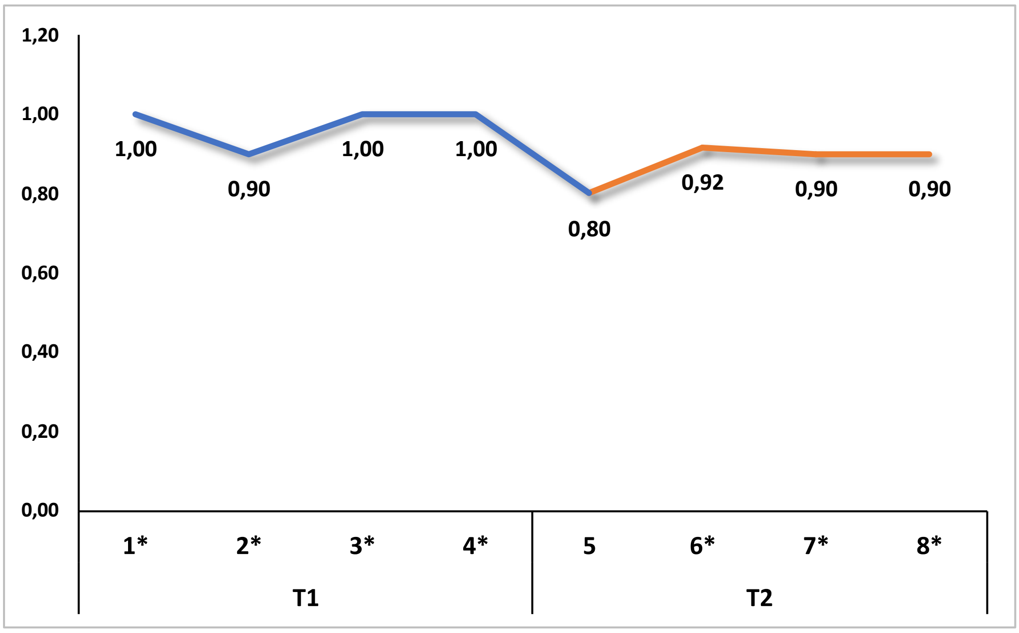

Fig. 16 Performance of L_02_22 in single sessions. According to the binomial test, most of the sessions were above chance level. *p<0.05

<u>Dog D_01_21</u> (Fig. 17, 18): The dog exhibited a calm demeanour during the training, proceeding gradually and cautiously towards the target and awaiting the verbal cue after hearing the sound sequence. The dog consistently refrained from exhibiting anticipatory behavior, resulting in no null trials throughout the training process.

In T1, the dog made some errors, and the number of correct trials in each individual session was significantly higher than expected by chance ($p<0.05$) in 2 sessions. However, when considering the overall performance in the number of correct trials, it was found to be above chance ($p<0.001$).

The results obtained in T2, were consistent with those in T1.

Regrettably, T3 was not conducted as the owner chose not to proceed due to personal engagements.

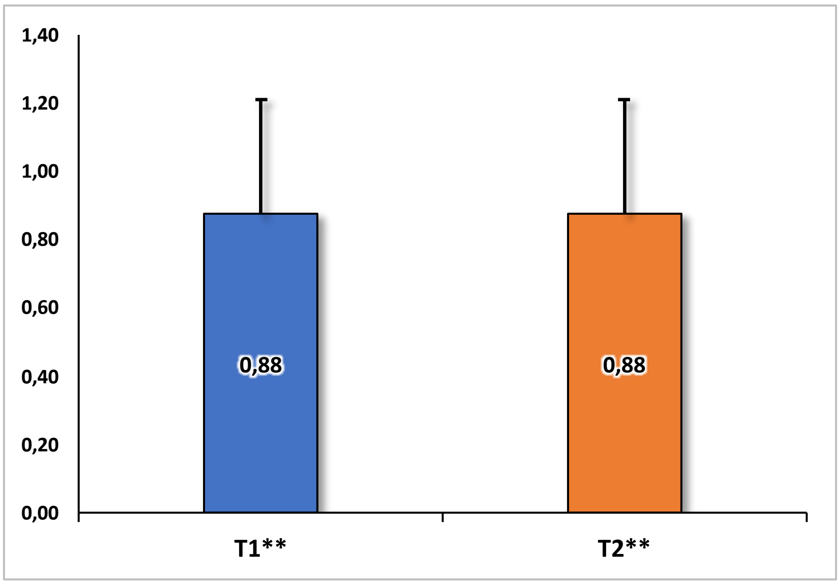

Fig. 17 Performance of D_01_21 in the training levels. According to the binomial test, T1 and T2 were above chance level. **$p<0.001$

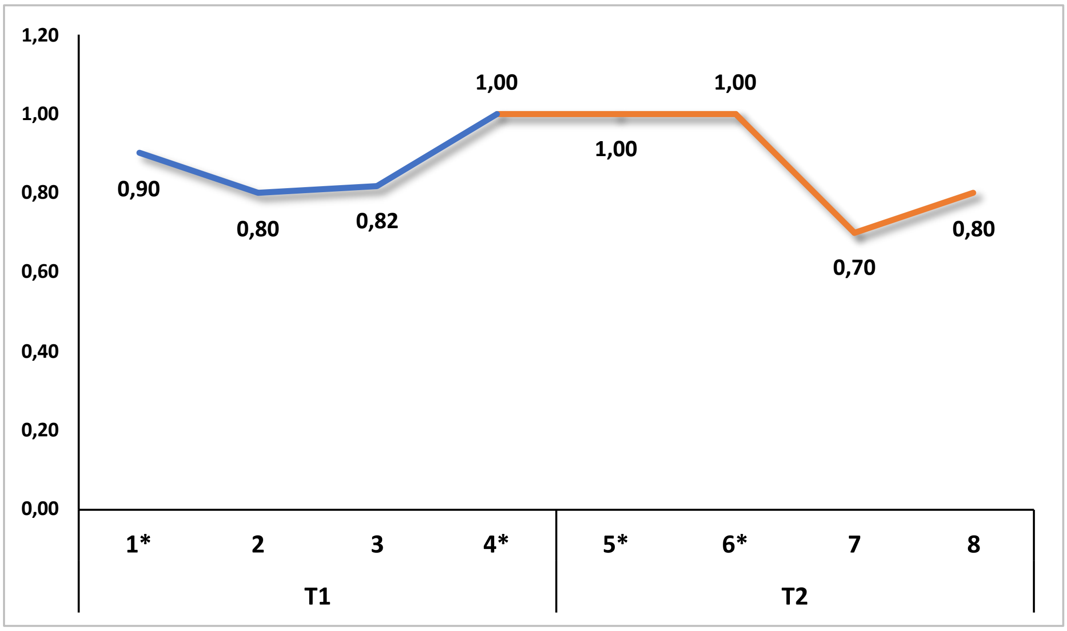

Fig. 18 Performance of D_01_21 in single sessions. According to the binomial test, half of the sessions were above chance level. *p<0.05

Dog LR_02_22 (Fig. 19, 20): The dog demonstrated a high level of attentiveness towards its owner and exhibited focused behavior in response to cues during the training sessions. The training effectively empowered the dog to initiate actions following the sound sequence without depending on verbal commands. Notably, the dog exhibited no anticipatory behavior and consistently remained within the study area, resulting in a complete absence of null responses.

During T1, the dog made some errors in its responses. However, when considering the overall performance in this training level, it significantly exceeded chance levels ($p<0.001$). Furthermore, in half of the individual sessions, the number of correct trials was significantly higher than expected by chance ($p<0.05$). Unfortunately, due to handler problems, the dog's progress halted at this training level.

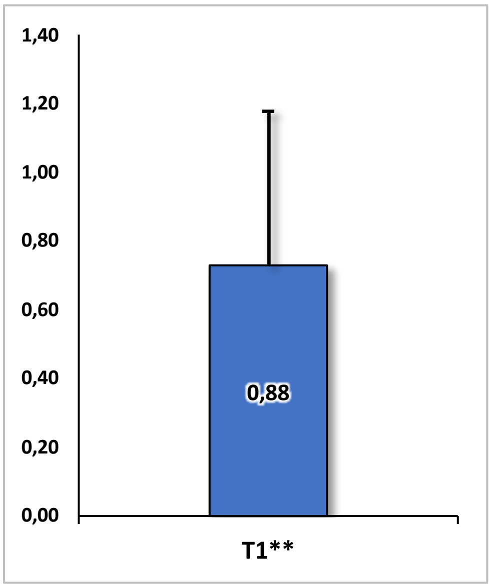

Fig. 19 Performance of LR_02_22 in the training level. According to the binomial test, the T1 performance was above chance level. **p<0.001

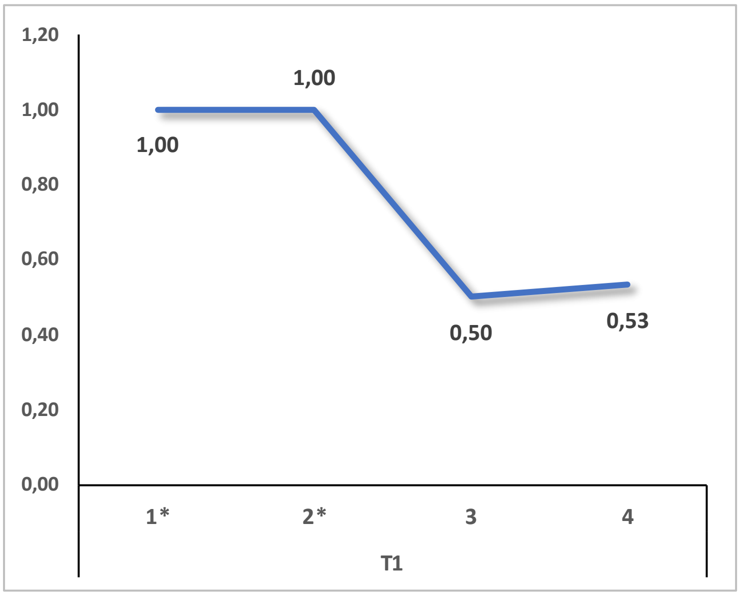

Fig. 20 Performance of LR_02_22 in single sessions. According to the binomial test, half of the sessions were above chance level. *p<0.05

Dog LC_01_22 (Fig. 21, 22): The dog exhibited thoughtful and calm behavior throughout the training sessions. It often relied on verbal cues before moving from its position after the sound sequence was played.

During T1, the dog performed well, making only one mistakes. The number of correct responses in three out of four sessions was significantly higher than expected by chance ($p<0.05$). Additionally, one session showed a tendency towards significance ($p=0.065$). The overall performance in this training level, the number of correct responses was also significantly above chance ($p<0.001$).

Unfortunately, the dog's progress was halted at level T1 due to handler problems.

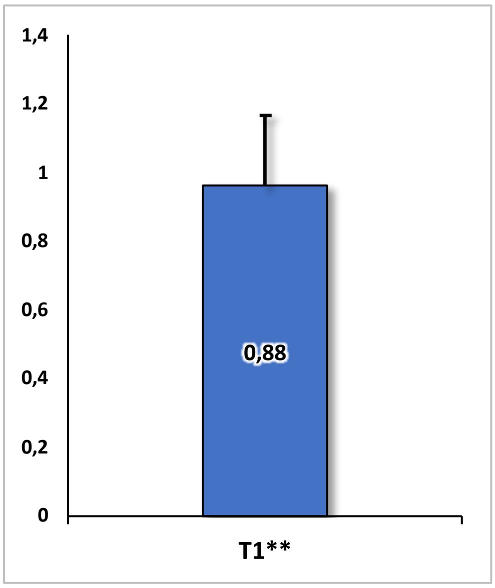

Fig. 21 Performance of LC_01_22 in the training level. According to the binomial test, the T1 performance was above chance level. **$p<0.001$

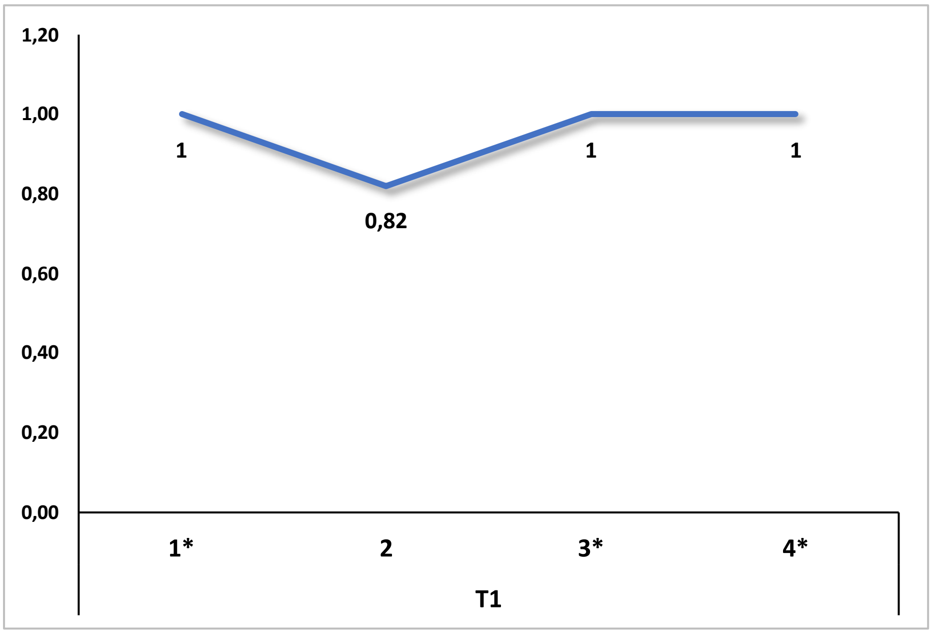

Fig. 22 Performance of LC_01_22 in single sessions. According to the binomial test, most of sessions were above chance level. *p<0.05

LONG-TRAINING

Dog BC_01_21 (Fig. 23, 24): This dog underwent training in the laboratory under the guidance of an experienced experimenter who had a strong familiarity with the dog. The dog exhibited exceptional focus on the task and showed enthusiasm during the training sessions. It consistently responded quickly and did not produce any null responses.

During T1, the dog's performance was flawless, consistently surpassing chance levels in the number of correct trials in each individual session ($p<0.05$), as well as in the overall training level ($p<0.001$).

In T2, consisting of 29 sessions, the dog achieved above-chance performance ($p<0.05$) in 20 sessions regarding the number of correct responses. The cumulative number of correct trials in this training level also yielded results above chance ($p<0.001$).

Nevertheless, in T3, the dog's performance remained at chance level, both in the individual sessions and the overall number of trials. As a result, the decision was made not to admit the dog to the test.

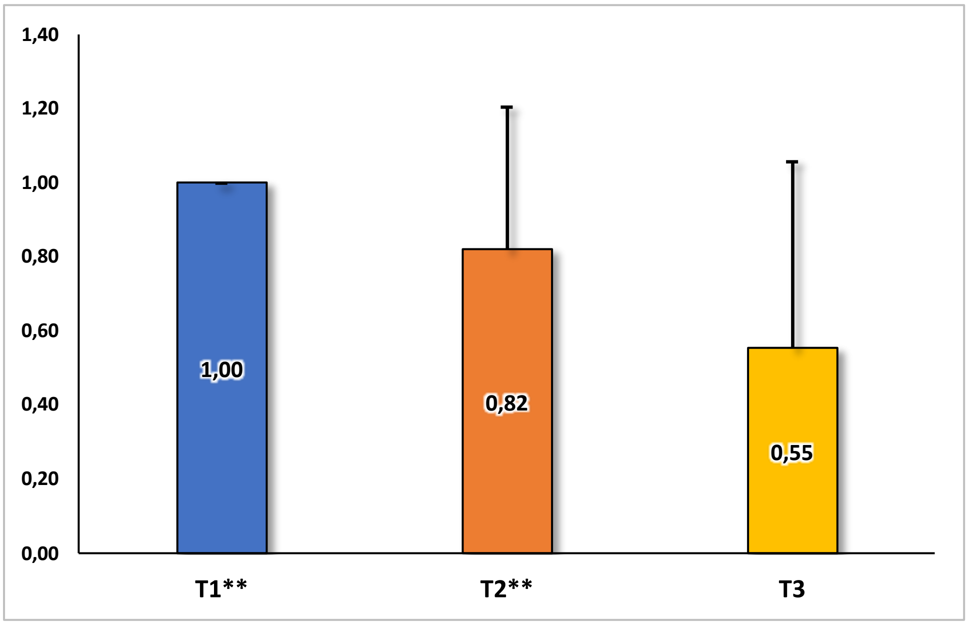

Fig. 23 Performance of BC_01_21 in the training levels. According to the binomial test, T1 and T2 were above chance level. **p<0.001

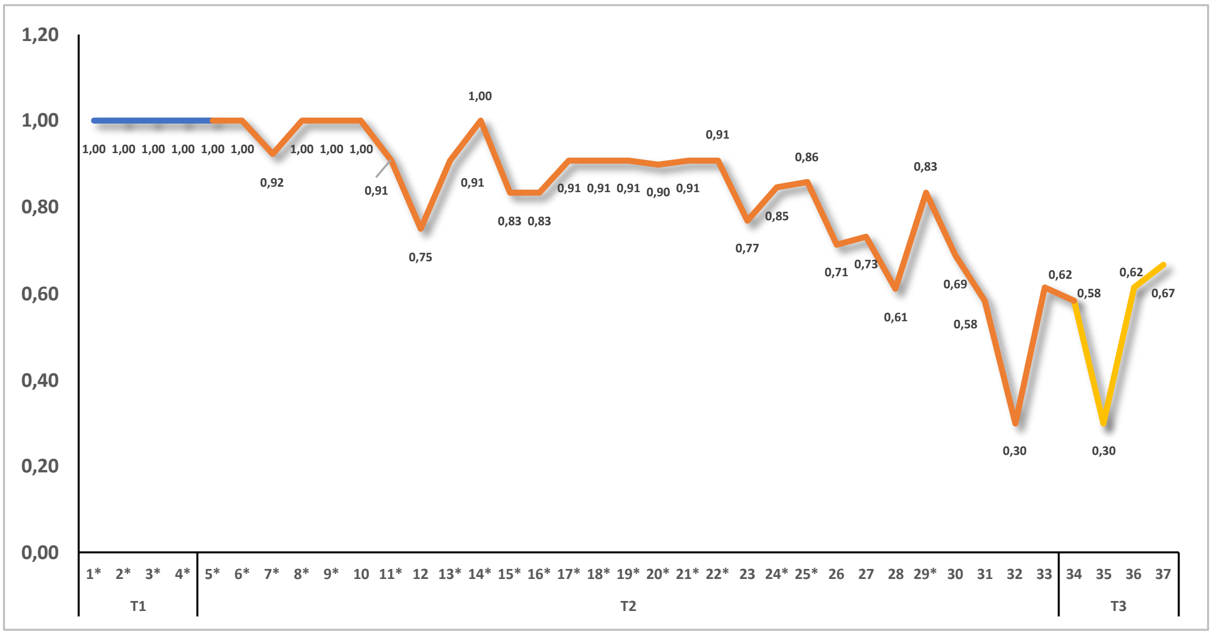

Fig. 24 Performance of BC_01_21 in single sessions. According to the binomial test, most of the sessions were above chance level. *p<0.05

Dog L_01_21 (Fig. 25, 26): The dog exhibited a high level of focus on the task and on its owner, who also happened to be one of the experimenters and a dog trainer. The dog responded swiftly and did not produce any null trials. However, it became evident early on that the dog relied heavily on visual cues. There were instances where it initiated the task without a verbal signal, although verbal prompting was occasionally necessary.

During T1, the dog consistently performed above chance levels in the number of correct trials in all individual sessions ($p<0.05$), as well as in the overall training level ($p<0.001$).

In T2, the dog’s performance varied, with some sessions being error-free while others included several mistakes. The number of correct trials exceeded chance levels ($p<0.05$) in 15 out of 24 sessions and throughout the entire training period ($p<0.001$).

Unfortunately, in T3, the dog's performance remained at chance level in both the individual sessions and the overall number of trials. Consequently, the dog was not deemed suitable for further testing.

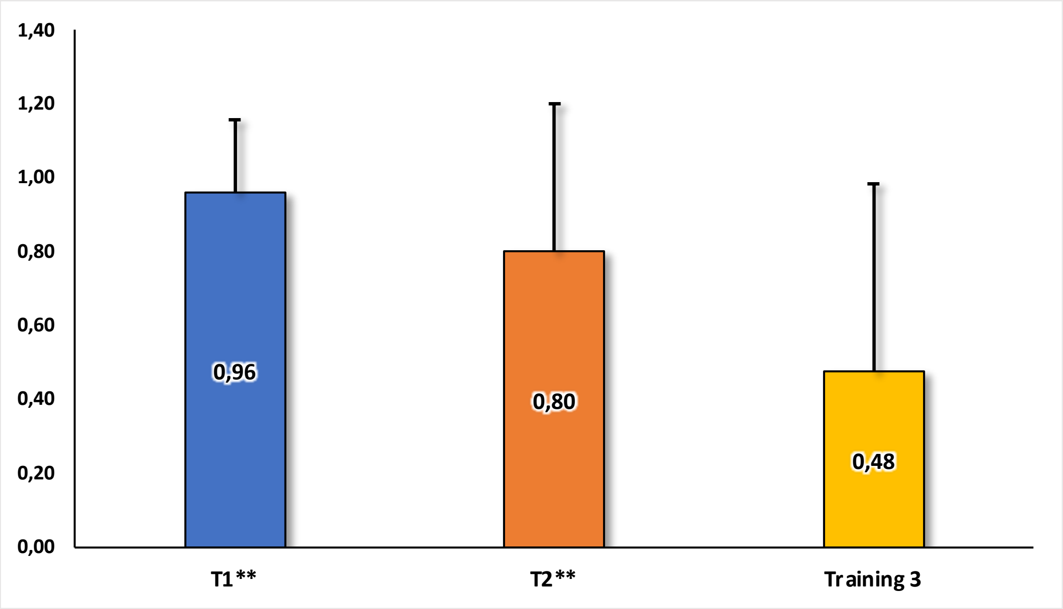

Fig. 25 Performance of L_01_21 in the training levels. According to the binomial test, T1 and T2 were above chance level. **$p<0.001$

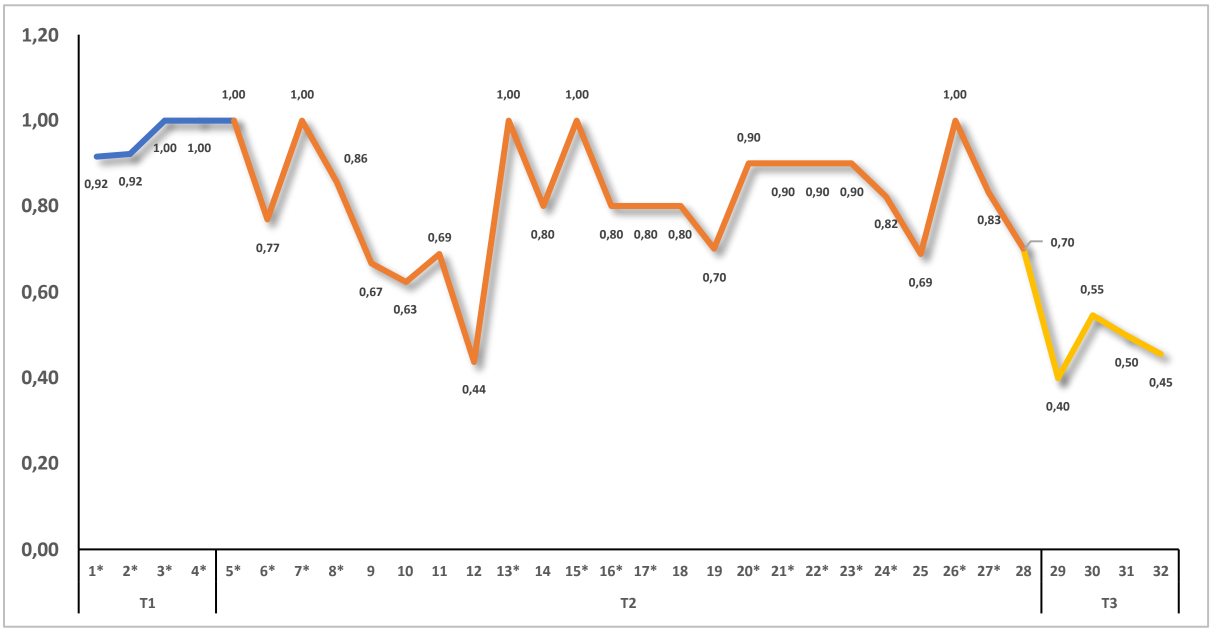

Fig. 26 Performance of L_01_21 in single sessions. According to the binomial test, most of the sessions were under chance level. *p<0.05

<u>Dog GS_01_21</u> (Fig. 27, 28): The dog exhibited a relatively low level of enthusiasm throughout the training sessions, but remained attentive and performed the tasks carefully. It maintained focus on the owner, who happened to be one of the experimenters, and executed controlled movements towards the bowl. Notably, the dog did not produce any null trials.

During T1, the dog consistently performed above chance levels (p<0.05) in the number of correct trials in all individual sessions making few mistakes. The overall performance in this training level was significantly above chance (p<0.001) when considering the number of correct trials.

In T2, the dog achieved generally high scores, surpassing chance levels (p<0.05) in the number of correct trials in 17 out of 25 sessions. Moreover, the overall performance in this training level, when considering the entire set of trials, was significantly above chance (p<0.001).

Unfortunately, in T3, the dog's performance remained at chance level both in the individual sessions and the cumulative number of trials in this training level. As a result, the dog was not admitted to the test.

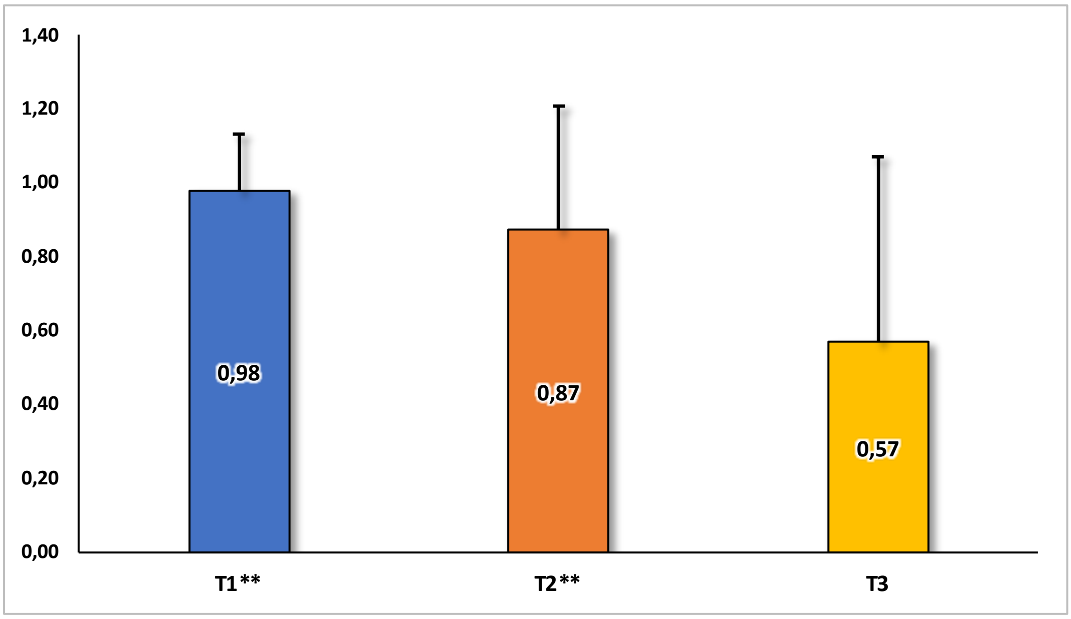

Fig. 27 Performance of GS_01_21 in the training levels. According to the binomial test, T1 and T2 were above chance level. **p<0.001

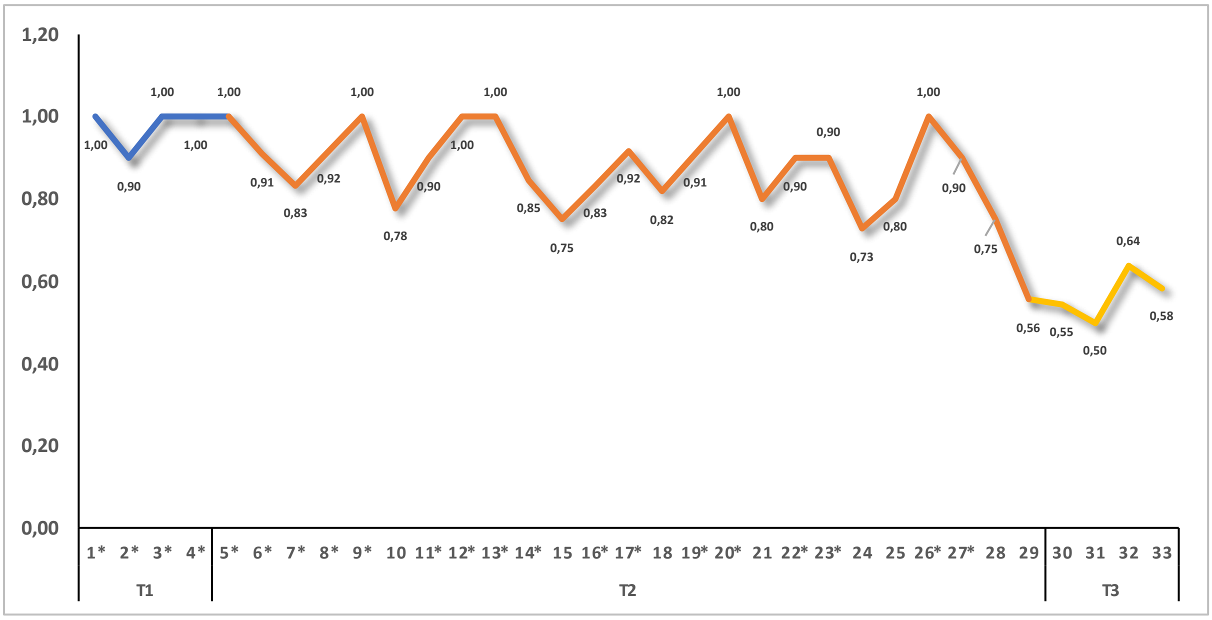

Fig. 28 Performance of GS_01_21 in single sessions. According to the binomial test, most of the sessions were above chance level. *p<0.05
